# Comorbidity clusters associated with newly treated type 2 diabetes mellitus: a Bayesian nonparametric analysis

**DOI:** 10.1038/s41598-022-24217-2

**Published:** 2022-11-30

**Authors:** Adrian Martinez-De la Torre, Fernando Perez-Cruz, Stefan Weiler, Andrea M. Burden

**Affiliations:** 1 Institute of Pharmaceutical Sciences, Department of Chemistry and Applied Biosciences, ETH Zurich, Vladimir-Prelog-Weg 1-5/10, 8093 Zurich, Switzerland; 2 Swiss Data Science Center, ETH Zurich and EPFL, Zurich, Switzerland; 3 Institute of Machine Learning, Department of Computer Science, ETH Zurich, Zurich, Switzerland

**Keywords:** Diabetes, Epidemiology

## Abstract

Type 2 diabetes mellitus (T2DM) is associated with the development of chronic comorbidities, which can lead to high drug utilization and adverse events. We aimed to identify common comorbidity clusters and explore the progression over time in newly treated T2DM patients. The IQVIA Medical Research Data incorporating data from THIN, a Cegedim database of anonymized electronic health records, was used to identify all patients with a first-ever prescription for a non-insulin antidiabetic drug (NIAD) between January 2006 and December 2019. We selected 58 chronic comorbidities of interest and used Bayesian nonparametric models to identify disease clusters and model their progression over time. Among the 175,383 eligible T2DM patients, we identified the 20 most frequent comorbidity clusters, which were comprised of 14 latent features (LFs). Each LF was associated with a primary disease (e.g., 98% of patients in cluster 2, characterized by LF2, had congestive heart failure [CHF]). The presence of certain LFs increased the probability of having another LF active. For example, LF2 (CHF) frequently appeared with LFs related to chronic kidney disease (CKD). Over time, the clusters associated with cardiovascular diseases, such as CHF, progressed rapidly. Moreover, the onset of certain diseases led to further complications. Our models identified established T2DM complications and previously unknown connections, thus, highlighting the potential for Bayesian nonparametric models to characterize complex comorbidity patterns.

## Introduction

Once patients are diagnosed with type 2 diabetes mellitus (T2DM), a constellation of chronic comorbidities might develop over time^1^. Common comorbidities are cardiovascular disease, diabetic retinopathy, peripheral neuropathy, and at later stages, chronic kidney disease (CKD) and musculoskeletal complications^2,3^. This implies that multimorbid T2DM patients have a high disease burden and are likely to experience a high degree of polypharmacy^4^. Understanding the development of comorbidities and identifying trajectory patterns may aid in developing more personalized management strategies. However, the evolution of chronic comorbidities in patients with T2DM is poorly understood.

With the growing availability of large electronic healthcare records and advances in machine learning, different statistical models have been used to find clusters of T2DM patients with similar diseases or comorbidity progression patterns. For instance, Aguado and colleagues used network analysis^5^ to identify comorbidity development following T2DM diagnosis, while Khan et al. utilized network analysis to predict the progression of diabetes^6^. A study by Ahlqvist et al.^7^, later replicated using clinical data by Dennis et al.^8^, identified five different subgroups of T2DM glycaemic progression using *k*-means hierarchical clustering based on six variables. Importantly, all these studies found that the clusters were associated with diabetic complications such as kidney disease or retinopathy. However, no study to date has examined changes in comorbidity clusters over time following the start of T2DM.

Modelling comorbidity progression can help clinicians understand and prevent poor health trajectories and potentially harmful polypharmacy. Previous studies have used latent class analysis (LCA) in healthcare data to broadly model multimorbidity trajectories of chronic diseases^9–11^. However, LCA models pose some limitations, such as they assume that the number of features is known and that the features follow a Gaussian distribution. Hence, we propose that adopting a Bayesian nonparametric model might help overcome these limitations as they allow data to be modelled in an unspecified number of latent features^12,13^. Only a few epidemiological studies have used this approach, for instance, in understanding comorbidities in patients with psychiatric disorders^14^ or suicide attempts^15^. However, Bayesian nonparametric models have never been used in electronic health records to understand T2DM disease progression.

Therefore, this study aimed to identify and describe the progression of common chronic comorbidities after T2DM onset using a Bayesian nonparametric model in a primary care electronic health records database.

## Results

### Patient cohort and characteristics

**Figure 1:**
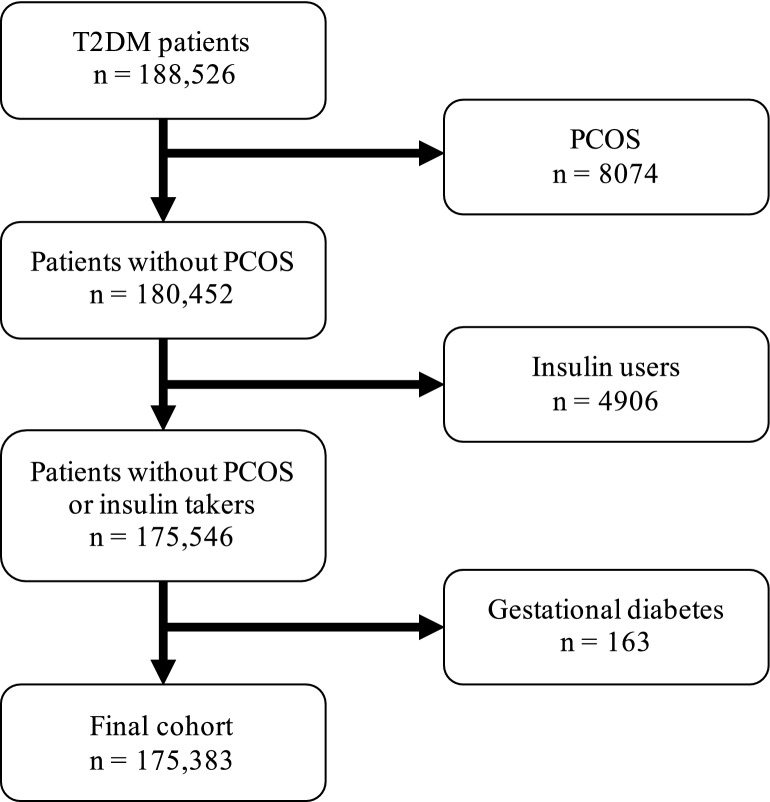
Flowchart of included patients. *T2DM* type 2 diabetes mellitus, *PCOS* polycystic ovarian syndrome.

Following exclusions, a total of 175,383 eligible T2DM patients were identified, Fig. 1. Table 1 provides the demographic characteristics of the patients at the index date, stratified by sex. There were 97,148 males and 78,235 females, with an average age of 60.6 years. The five most prevalent comorbidities at the index date were high blood pressure (38.1%), cancer (25.5%), osteoarthritis (19.8%), and anxiety and depression (17.2%).

**Table 1:** Demographic characteristics of 175,383 T2DM patients at index date (first NIAD prescription).

	Overall (N = 175,383)	Male (N = 97,148)	Female (N = 78,235)	SMD
Index age (mean (SD))	60.6 (14.1)	60.7 (12.8)	60.4 (15.6)	0.03
**Smoking (%)**				0.33
Current	60,206 (34.5)	39,227 (40.6)	20,979 (26.9)	
Never	83,719 (48.0)	39,715 (41.1)	44,004 (56.5)	
Previous	30,641 (17.6)	17,729 (18.3)	12,912 (16.6)	
Unknown	< 6	< 6	< 6	
**Alcohol consumption (%)**				0.40
Current	7368 (4.5)	4342 (4.8)	3026 (4.2)	
Never	38,687 (23.7)	14,679 (16.2)	24,008 (33.2)	
Previous	116,823 (71.7)	71,470 (79.0)	45,353 (62.6)	
Unknown	29 (0.0)	16 (0.0)	13 (0.0)	
**BMI (mean (SD))**	32.54 (7.0)	31.84 (6.2)	33.39 (7.7)	0.22
**Follow-up time in years (mean (SD))**	7.4 (3.9)	7.4 (3.9)	7.3 (3.9)	0.03
**Comorbidities ever-before (%)**				
Cancer	44,670 (25.5)	21,275 (21.9)	23,395 (29.9)	0.18
Hypothyroidism	13,940 (7.9)	3234 (3.3)	10,706 (13.7)	0.38
Pure hypercholesterolaemia	28,287 (16.1)	16,118 (16.6)	12,169 (15.6)	0.03
Obesity	20,051 (11.4)	8760 (9.0)	11,291 (14.4)	0.17
Anxiety & other	30,167 (17.2)	12,273 (12.6)	17,894 (22.9)	0.27
Neuropathy	1278 (0.7)	769 (0.8)	509 (0.7)	0.02
Primary open-angle glaucoma	6072 (3.5)	3252 (3.3)	2820 (3.6)	0.01
Senile cataract	8208 (4.7)	3775 (3.9)	4433 (5.7)	0.08
Deafness	14,323 (8.2)	8487 (8.7)	5836 (7.5)	0.05
High blood pressure	66,828 (38.1)	36,341 (37.4)	30,487 (39.0)	0.03
Angina pectoris	11,512 (6.6)	7400 (7.6)	4112 (5.3)	0.10
Atrial fibrillation	12,342 (7.0)	7161 (7.4)	5181 (6.6)	0.03
Congestive heart failure	5016 (2.9)	3135 (3.2)	1881 (2.4)	0.05
Intermittent claudication	5486 (3.1)	3415 (3.5)	2071 (2.6)	0.05
Chronic bronchitis	2848 (1.6)	1446 (1.5)	1402 (1.8)	0.02
Irritable bowel syndrome	17,194 (9.8)	6496 (6.7)	10,698 (13.7)	0.23
Chronic liver disease	4345 (2.5)	2407 (2.5)	1938 (2.5)	0.00
Chronic kidney disease	1070 (0.6)	608 (0.6)	462 (0.6)	0.00
Psoriasis or eczema	10,297 (5.9)	5594 (5.8)	4703 (6.0)	0.01
Osteoarthritis	34,683 (19.8)	15,915 (16.4)	18,768 (24.0)	0.19
Arthropathy	9991 (5.7)	4298 (4.4)	5693 (7.3)	0.12
Cervical spondylosis	13,025 (7.4)	6392 (6.6)	6633 (8.5)	0.07
Osteoporosis	7171 (4.1)	2058 (2.1)	5113 (6.5)	0.22

SMD, Standardized mean difference.

All identified comorbidities were identified via read codes, as recorded anytime on or before the index date, defined as the first prescribed non-insulin antidiabetic drug (NIAD).

### Comorbidity cluster identification and characteristics

From the initial list of 58 chronic conditions, we selected a total of 23 conditions that had a prevalence higher than 1.0% to avoid numerical and convergence problems of the Bayesian nonparametric model. The selected chronic comorbidities are shown in Supplementary Table S1.

We found 14 different latent features, of which the first one, the bias term, was active for every patient. The 14 latent features resulted in 385 clusters, each corresponding to a unique combination of the latent features. Table 2 provides an overview of the 20 most common clusters and the top three most prevalent conditions associated with each. Except for cluster 1, which includes the bias term (i.e., latent feature 1), most of the clusters were represented by one highly prevalent chronic disease with other additional diseases having elevated O/E ratios. For example, the second cluster, which had latent feature 2 active, was strongly associated with congestive heart failure (CHF), Table 2. Overall, 98% of the patients in the second cluster had CHF, and the O/E ratio was 43.4. Additionally, once a patient developed CHF, the probability of concomitantly having atrial fibrillation and senile cataract was increased 7.0- and 2.3-fold, respectively, as seen in the corresponding O/E ratios. The third cluster was mainly composed of patients with hypothyroidism, while the fourth and fifth were characterized by patients with osteoporosis and obesity, respectively.

**Table 2:** Description of the three most prevalent conditions for the first 20 clusters.

Cluster	Latent features	Recorded comorbidities	Count	N cluster	Count prop (%)	Total disease	Total dis. prop (%)	O/E ratio	
1	LF1	High blood pressure	8396	147,816	5.7	10,660	6.1	0.9	
1	LF1	Pure hypercholesterolaemia	4570	147,816	3.1	5938	3.4	0.9	
1	LF1	Chronic liver disease	3437	147,816	2.3	4505	2.6	0.9	
2	LF1 LF2	Congestive heart failure	2711	2766	98.0	3962	2.3	43.4	
2	LF1 LF2	Atrial fibrillation	776	2766	28.1	7000	4.0	7.0	
2	LF1 LF2	Senile cataract	281	2766	10.2	7709	4.4	2.3	
3	LF1 LF3	Hypothyroidism	2535	2572	98.6	3260	1.9	53.0	
3	LF1 LF3	Irritable bowel syndrome	118	2572	4.6	5019	2.9	1.6	
3	LF1 LF3	Anxiety and other	92	2572	3.6	4115	2.3	1.5	
4	LF1 LF4	Osteoporosis	2323	2350	98.9	3233	1.8	53.6	
4	LF1 LF4	Senile cataract	269	2350	11.4	7709	4.4	2.6	
4	LF1 LF4	Irritable bowel syndrome	148	2350	6.3	5019	2.9	2.2	
5	LF1 LF5	Obesity	2235	2252	99.2	2879	1.6	60.5	
5	LF1 LF5	Pure hypercholesterolaemia	185	2252	8.2	5938	3.4	2.4	
5	LF1 LF5	Chronic liver disease	121	2252	5.4	4505	2.6	2.1	
6	LF1 LF6	Intermittent claudication	1855	1871	99.1	2653	1.5	65.5	
6	LF1 LF6	Atrial fibrillation	153	1871	8.2	7000	4.0	2.1	
6	LF1 LF6	Senile cataract	153	1871	8.2	7709	4.4	1.9	
7	LF1 LF7	Primary open-angle glaucoma	1789	1795	99.7	2330	1.3	75.0	
7	LF1 LF7	Senile cataract	289	1795	16.1	7709	4.4	3.7	
7	LF1 LF7	Osteoarthritis	143	1795	8.0	8954	5.1	1.6	
8	LF1 LF9	Arthropathy	1531	1532	99.9	2168	1.2	80.8	
8	LF1 LF9	Osteoarthritis	290	1532	18.9	8954	5.1	3.7	
8	LF1 LF9	Anxiety and other	70	1532	4.6	4115	2.3	2.0	
9	LF1 LF8	Chronic bronchitis	1494	1496	99.9	2260	1.3	77.5	
9	LF1 LF8	Deafness	95	1496	6.4	5261	3.0	2.1	
9	LF1 LF8	Senile cataract	134	1496	9.0	7709	4.4	2.0	
10	LF1 LF10	Psoriasis or eczema	1477	1481	99.7	1975	1.1	88.6	
10	LF1 LF10	Osteoarthritis	144	1481	9.7	8954	5.1	1.9	
10	LF1 LF10	Chronic liver disease	71	1481	4.8	4505	2.6	1.9	
11	LF1 LF12	Cervical spondylosis	1369	1371	99.9	1937	1.1	90.4	
11	LF1 LF12	Osteoarthritis	204	1371	14.9	8954	5.1	2.9	
11	LF1 LF12	Irritable bowel syndrome	97	1371	7.1	5019	2.9	2.5	
12	LF1 LF11	Neuropathy	1350	1350	100.0	1964	1.1	89.3	
12	LF1 LF11	Chronic liver disease	65	1350	4.8	4505	2.6	1.9	
12	LF1 LF11	Irritable bowel syndrome	70	1350	5.2	5019	2.9	1.8	
13	LF1 LF14	Angina pectoris	1253	1255	99.8	1773	1.0	98.8	
13	LF1 LF14	Deafness	80	1255	6.4	5261	3.0	2.1	
13	LF1 LF14	Atrial fibrillation	105	1255	8.4	7000	4.0	2.1	
14	LF1 LF13	Chronic kidney disease	1227	1228	99.9	1869	1.1	93.8	
14	LF1 LF13	Atrial fibrillation	109	1228	8.9	7000	4.0	2.2	
14	LF1 LF13	Deafness	81	1228	6.6	5261	3.0	2.2	
15	LF1 LF13 LF2	Chronic kidney disease	135	135	100.0	1869	1.1	93.8	
15	LF1 LF13 LF2	Congestive heart failure	135	135	100.0	3962	2.3	44.3	
15	LF1 LF13 LF2	Atrial fibrillation	45	135	33.3	7000	4.0	8.4	
16	LF1 LF8 LF2	Chronic bronchitis	126	126	100.0	2260	1.3	77.6	
16	LF1 LF8 LF2	Congestive heart failure	125	126	99.2	3962	2.3	43.9	
16	LF1 LF8 LF2	Atrial fibrillation	49	126	38.9	7000	4.0	9.7	
17	LF1 LF4 LF2	Osteoporosis	117	117	100.0	3233	1.8	54.3	
17	LF1 LF4 LF2	Congestive heart failure	117	117	100.0	3962	2.3	44.3	
17	LF1 LF4 LF2	Atrial fibrillation	29	117	24.8	7000	4.0	6.2	
18	LF1 LF3 LF2	Hypothyroidism	103	103	100.0	3260	1.9	53.8	
18	LF1 LF3 LF2	Congestive heart failure	103	103	100.0	3962	2.3	44.3	
18	LF1 LF3 LF2	Atrial fibrillation	35	103	34.0	7000	4.0	8.5	
19	LF1 LF6 LF2	Intermittent claudication	101	101	100.0	2653	1.5	66.1	
19	LF1 LF6 LF2	Congestive heart failure	101	101	100.0	3962	2.3	44.3	
19	LF1 LF6 LF2	Atrial fibrillation	32	101	31.7	7000	4.0	7.9	
20	LF1 LF14 LF2	Angina pectoris	96	96	100.0	1773	1.0	98.9	
20	LF1 LF14 LF2	Congestive heart failure	96	96	100.0	3962	2.3	44.3	
20	LF1 LF14 LF2	Atrial fibrillation	25	96	26.0	7000	4.0	6.5	

Count, numbers of patients with that disease in that cluster; N cluster, total number of individuals within that cluster; Count prop, proportion of patients who have that disease within a cluster; Total disease, overall number of patients with that disease; Total dis. Prop, overall proportion with that disease; O/E ratio, observed to expected ratio.

All identified comorbidities were identified via read codes, as recorded anytime on or before the index date, defined as the first prescribed non-insulin antidiabetic drug (NIAD).

From cluster 15 on, we observed that the clusters resulted from combining two or more latent features, Table 2. Hence, patients had two distinct primary diseases along with other secondary comorbidities. For instance, cluster 15 was composed of patients who all (100%) had chronic kidney disease (CKD) and CHF, while one-third (33.3%) had atrial fibrillation. All three top conditions also had elevated O/E ratios of 93.8, 44.3, and 8.4, respectively. In cluster 16, all (100%) patients had chronic bronchitis, 99.2% also had CHF, and 38.9% had atrial fibrillation. Again, elevated O/E ratios were identified for the three top conditions. The complete list of comorbidities identified per cluster is provided in Supplementary Table S2.

Sex differences between clusters were also identified, as shown in Supplementary Figure S1. For example, clusters 2 and 6 (cardiovascular disease clusters) were more heavily dominated by males, as evidenced by the lower proportion of females within the clusters (34.8% and 33.1% female, respectively). Similarly, the O/E ratios for the gender distribution were below 1.0 in both clusters (e.g., 0.70 and 0.78 for clusters 2 and 6, respectively). Conversely, other clusters were female-dominated. For example, clusters 3 and 4 (hypothyroidism and osteoporosis) consisted of 57.8% and 71.3% females, respectively, and the sex O/E ratios were 1.3 and 1.6, respectively), Supplementary Figure S1.

In Table 3, we present the probability of presenting at least one of the latent features active, either in combination with other latent features or as a single feature. We found that 84.3% of the individuals had only the bias term, latent feature 1, active. Moreover, certain comorbidities were more likely to appear than others. For instance, latent feature 2, corresponding to CHF, was the feature with the highest probability of being active, either in combination with other features (2.3%) or as a single feature (1.6%). The least likely features to be active, either in combination with others or as a single feature, were latent features 13 and 14, associated with chronic kidney disease and angina pectoris, respectively, as shown in Table 3. Hence, having CHF and subsequent comorbidities was more likely than having CKD with other comorbidities.

**Table 3:** Probabilities (%) of possessing at least one latent feature or a single feature.

Latent feature	Total (%)	Single feature (%)	Dominant feature
1	100	84.28	High blood pressure
2	2.30	1.58	Congestive heart failure
3	1.89	1.47	Hypothyroidism
4	1.87	1.34	Irritable bowel syndrome
5	1.65	1.28	Obesity
6	1.53	1.07	Intermittent claudication
7	1.34	1.02	Primary open-angle glaucoma
8	1.29	0.85	Arthropathy
9	1.24	0.87	Chronic bronchitis
10	1.13	0.84	Psoriasis or eczema
11	1.12	0.77	Cervical spondylosis
12	1.11	0.78	Neuropathy
13	1.07	0.70	Angina pectoris
14	1.01	0.72	Chronic kidney disease

The total column represents the total number of patients with the individual latent feature. While the single feature column represents the number of patients with at least that specific latent feature active. Each latent feature (LF) corresponds to a group of comorbidities. However, for each latent feature, one dominant comorbidity was identified (see Table 2).

The probability of having at least two latent features active is presented in Table 4. We found that the empirical probability of two latent features was around twice as large as the product probabilities, indicating that an active latent feature was associated with an increased probability of having another latent feature active. For instance, the empirical probability of having latent feature 2 active, which is dominated by a high prevalence of CHF, and latent feature 4, associated with osteoporosis, was 0.11%, which was 2.5 times higher than the product probability of 0.04%.

**Table 4:** Probabilities of possessing at least two latent features.

Latent features	1 (%)	2 (%)	3 (%)	4 (%)	5 (%)	6 (%)	7 (%)	8 (%)	9 (%)	10 (%)	11 (%)	12 (%)	13 (%)	14 (%)
1		2.30	1.89	1.87	1.65	1.53	1.34	1.29	1.24	1.13	1.12	1.11	1.07	1.01
2	2.30		0.04	0.04	0.04	0.04	0.03	0.03	0.03	0.03	0.03	0.03	0.02	0.02
3	1.89	0.08		0.04	0.03	0.04	0.03	0.02	0.02	0.02	0.02	0.02	0.02	0.02
4	1.87	0.11	0.07		0.03	0.06	0.05	0.02	0.02	0.03	0.04	0.06	0.02	0.02
5	1.65	0.07	0.04	0.03		0.03	0.02	0.02	0.02	0.03	0.02	0.04	0.02	0.02
6	1.53	0.10	0.03	0.03	0.03		0.02	0.02	0.02	0.02	0.02	0.02	0.02	0.02
7	1.34	0.05	0.04	0.02	0.03	0.04		0.02	0.02	0.02	0.01	0.03	0.01	0.01
8	1.29	0.11	0.04	0.08	0.04	0.06	0.02		0.04	0.03	0.03	0.03	0.01	0.01
9	1.24	0.07	0.03	0.05	0.05	0.04	0.03	0.02		0.04	0.03	0.04	0.01	0.01
10	1.13	0.03	0.03	0.02	0.02	0.02	0.02	0.01	0.01		0.01	0.03	0.01	0.01
11	1.12	0.04	0.03	0.02	0.03	0.07	0.03	0.01	0.01	0.03		0.03	0.01	0.01
12	1.11	0.04	0.03	0.02	0.02	0.03	0.01	0.01	0.01	0.01	0.01		0.01	0.01
13	1.07	0.12	0.05	0.05	0.03	0.04	0.02	0.03	0.03	0.02	0.03	0.03		0.01
14	1.01	0.08	0.02	0.04	0.03	0.04	0.02	0.03	0.02	0.02	0.03	0.03	0.02	

Empirical probabilities were directly extracted from the observed latent matrix.

Product probabilities were calculated from the individual latent feature probabilities, as reported in Table 3.

The elements below the diagonal correspond to the empirical probability, and the elements above the diagonal correspond to the product probability.

Additionally, we also saw that some diseases increased the probability of having concomitantly other diseases. Having a given feature active led to an increased probability of having another one active, Table 5. For example, latent feature 2 appeared frequently with features 4, 6, 8, 13, and 14. Therefore, this would indicate that osteoporosis, intermittent claudication, arthropathy, angina pectoris, and CKD were commonly associated with CHF in our T2DM cohort.

**Table 5:** Empirical probabilities of possessing at least latent features ${k}_{1}$ and ${k}_{2}$ given that ${k}_{1}$ is active.

${k}_{1}$	${k}_{2}$													
	1 (%)	2 (%)	3 (%)	4 (%)	5 (%)	6 (%)	7 (%)	8 (%)	9 (%)	10 (%)	11 (%)	12 (%)	13 (%)	14 (%)
1	100.00	2.30	1.89	1.87	1.65	1.53	1.34	1.29	1.24	1.13	1.12	1.11	1.07	1.01
2	100.00	100.00	3.55	4.64	2.88	4.17	2.33	4.72	2.83	1.37	1.94	1.71	5.02	3.53
3	100.00	4.31	100.00	3.44	2.08	2.32	2.08	2.20	1.72	1.51	1.81	1.75	2.56	1.27
4	100.00	5.71	3.48	100.00	1.56	2.96	2.53	4.31	2.93	1.83	2.38	3.08	2.81	2.14
5	100.00	4.00	2.38	1.76	100.00	2.10	2.07	2.34	2.79	1.79	1.86	2.48	1.69	1.83
6	100.00	6.28	2.88	3.63	2.28	100.00	2.36	3.74	2.43	1.57	4.75	2.24	2.69	2.88
7	100.00	4.01	2.95	3.54	2.56	2.69	100.00	1.84	2.01	1.79	2.05	1.96	1.75	1.20
8	100.00	8.39	3.22	6.23	3.00	4.42	1.90	100.00	3.22	2.47	2.65	2.52	2.61	2.08
9	100.00	5.25	2.63	4.42	3.73	3.00	2.17	3.36	100.00	3.27	2.30	3.27	2.21	1.75
10	100.00	2.78	2.53	3.03	2.63	2.12	2.12	2.83	3.59	100.00	2.27	2.53	1.87	1.92
11	100.00	3.97	3.05	3.97	2.75	6.46	2.44	3.05	2.54	2.29	100.00	2.95	2.70	2.34
12	100.00	3.56	2.99	5.21	3.71	3.09	2.37	2.94	3.66	2.58	2.99	100.00	2.27	2.53
13	100.00	10.80	4.54	4.92	2.62	3.85	2.19	3.15	2.57	1.98	2.83	2.35	100.00	1.92
14	100.00	8.00	2.37	3.94	2.99	4.34	1.58	2.65	2.14	2.14	2.59	2.76	2.03	100.00

To compute the empirical probabilities, we looked at the number of patients that had latent feature ${k}_{1}$ active and counted how many of them also have latent feature ${k}_{2}$ active.

Complementarily, we compared the three main clusters associated with cardiovascular disease, Supplementary Table S3. We present the proportion of patients with each comorbidity overall and within the three clusters. Additionally, the O/E ratios by cluster are provided. Additionally, we compared the proportions for each comorbidity across clusters, using cluster 2 as the comparator. Cluster 2 was characterized by latent feature 2, CHF, and was associated with a higher prevalence of atrial fibrillation and senile cataract. However, when latent feature 13 was also active, cluster 15, a slight shift was observed. Here, 100% of the individuals had CHF and CKD. Moreover, most of the O/E ratios increased in this cluster compared to cluster 2; except for deafness, irritable bowel syndrome, anxiety, and chronic liver disease. Similarly, when latent features 2 and 8 were active in cluster 16, 100% of the patients had chronic bronchitis, and again most of the O/E ratios were increased.

Additionally, comparing baseline characteristics stratified by cluster, similar relationships were found, Supplementary Table S4. For instance, in cluster 2, atrial fibrillation and angina pectoris were highly prevalent at the index date, 19.2% and 16.8%, respectively. Nonetheless, CHF had a low prevalence at baseline, < 1%, suggesting that CHF might develop after atrial fibrillation.

### Evolution of clusters over time

**Figure 2:**
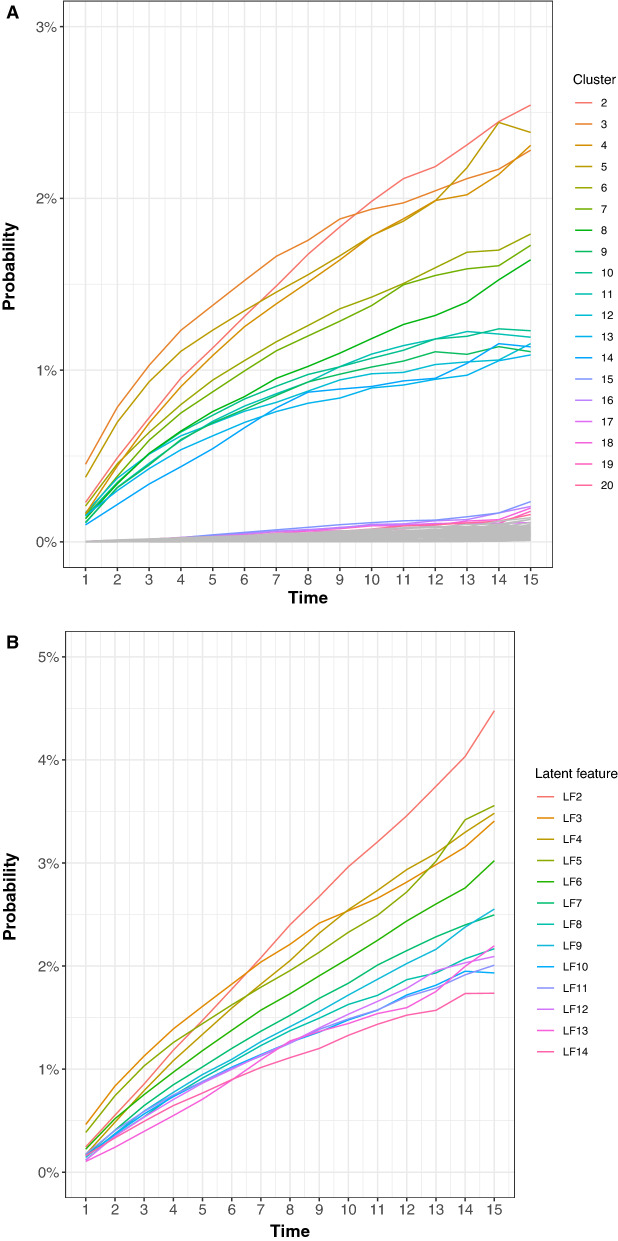
(**A**) Probability of belonging to each cluster over time. Note that some clusters increased at a higher rate compared to others. More information on the cluster characteristics can be found in Table 2. (**B**) Evolution of active latent features over time. Latent feature 1 is not depicted as it is always active.

In Fig. 2A, we visualize the progression of the top 20 clusters by estimating the proportion of patients belonging to the individual clusters over time, while the probability of the 14 individual latent features being active over time is provided in Fig. 2B. We found that the proportion of people belonging to each cluster increased over time, Fig. 2A.

Similarly, looking at the 14 individual latent features in Fig. 2B, we found that the probability of having a given latent feature active increased over time, except for the first latent feature (not shown), which was always active with a constant probability of 1. We observe that the proportion of patients with latent feature 2, which was associated with a high prevalence of CHF, increased at a higher rate compared to the other features. We also see an increase in the prevalence of latent feature 4, osteoporosis, over time Fig. 2B.

The network analysis that depicts the transition between the top 20 clusters over time is provided in Supplementary Figure S2. Overall, patients tended to remain in the same cluster over time. However, transitions from the first (latent feature 1 active) cluster to the other 14 (characterized by a single latent feature) were the most frequent. We further note that patients in clusters 5, 7, 8, 10–12 did not transition to other more complex clusters over time. However, a transition into cluster 2, was associated with further transitions into clusters 15–20, which were characterized by the presence of 2 active latent features.

## Discussion

This study confirmed the potential of using a large electronic healthcare database to identify clusters of chronic disease comorbidities among patients with newly treated T2DM. This is the first analysis that applied a Bayesian nonparametric model to real-world electronic medical records to identify distinct comorbidity clusters and disease progression patterns based on hidden latent features. In our case example of patients with T2DM, we could identify 14 different latent features that were strongly associated with a primary disease. Importantly, we identified comorbidity patterns consistent with the literature, pointing to the applicability of this approach in medical data. Thus, we found that Bayesian nonparametric models are a powerful tool to use in electronic health records to identify unique comorbidity clusters and health trajectories.

Understanding disease progression in T2DM patients is paramount to preventing new disease onset, optimizing treatment strategies, reducing polypharmacy, and increasing the safety and effectiveness of therapeutic options. However, due to the complexity of comorbidity patterns, there is a lack of understanding of patterns or trajectories. Previous studies have modelled T2DM progression in electronic health records using different approaches, including network modelling^6^, naïve Bayes, support vector machines, random forests, and gradient boosted trees^16,17^, or by using typical and atypical disease trajectory analysis^18^. Although these approaches can shed some light on the disease progression and comorbidities development, they might not be able to capture relationships between hidden or unknown risk factors. While using latent feature models can overcome important shortcomings of the aforementioned approaches, most models require pre-specifying the number of latent features to be retrieved. Consequently, they might not perform very well in the presence of binary matrices and might lack interpretability because latent features might extend over the real line^14,15^.

The results of our study identify that a Bayesian nonparametric model is a novel approach for studying chronic comorbidity progression. Bayesian nonparametric models overcome the limitations of traditional latent feature models as they can automatically infer the number of binary latent features from the data^19^. Using this approach, we found that the development of certain comorbidities can lead to a dramatic increase in the probability of developing other conditions over time. For example, in our analysis, once a patient with T2DM develops CHF, their probability of being diagnosed with atrial fibrillation increases, as seen in cluster 2. We also found that patients with hypothyroidism had an elevated likelihood of being diagnosed with irritable bowel syndrome, anxiety, and neurotic disorders increase^20,21^. While previous literature has found individual associations between hypothyroidism, irritable bowel syndrome, and anxiety^22,23^, the link between these as a common cluster, particularly among patients with T2DM, has not been previously identified. Therefore, our models could identify hidden (or previously unknown) connections between the diseases that form each cluster.

While our models identified unique comorbidity clusters, the predicted posterior probabilities were consistent with the known progression of T2DM. For instance, we found that all latent features, especially latent feature 2, which was associated with cardiovascular events, steadily increased over time. Conversely, the posterior probability for the baseline cluster, only latent feature 1 active, decreased over time. These results are in line with previous literature. For instance, Khan et al. found that cardiovascular conditions such as cardiac arrhythmias or hypertension were the most prevalent diseases appearing after T2DM onset^6^. Similarly, Oh and colleagues identified hyperlipidaemia and hypertension as frequent comorbidities after T2DM diagnosis^18^. Hence, after T2DM onset, the probability of developing certain comorbidities increases over the course of the disease.

Although our study was population-based and applied Bayesian nonparametric models, which overcome many of the limitations found in previous work, there are remaining limitations that must be considered when interpreting the results of this study. Firstly, we only looked at a specific subset of 23 different chronic comorbidities. Thus, we might have missed some patterns in the data. Moreover, we did not include acute outcomes in our list of comorbidities. We acknowledge that chronic diseases can increase the risk of experiencing an acute event, and acute events can also trigger or accelerate the onset of new chronic conditions. Therefore, future research could assess if similar trajectories are found when incorporating acute events or the impact of the chronic disease clusters on the onset of new acute outcomes.

In addition, since comorbidities were coded as binary variables and remained active after the first diagnosis, we might have missed different severity levels that a chronic disease might have had. Moreover, we did not include pharmacological treatments, which can impact the onset/delay of new comorbidities or alter the current disease status.

Cancer is a very complex and heterogeneous disease that requires thorough medical attention. In our analysis, we grouped all cancer diagnoses as a single disease for interpretability. Nonetheless, we might have missed links between different cancer types and comorbidity clusters, particularly those more commonly associated with T2DM (e.g., pancreatic or gastric cancer). Therefore, future studies may consider using Bayesian nonparametric models to investigate comorbidity clusters associated with specific cancer diagnoses to generate new hypotheses in diabetes patients.

In this population-based study of patients with T2DM, we could confirm the potential of using a Bayesian nonparametric model to identify distinct patient clusters. Our models found results consistent with the literature (e.g., growing prevalence of cardiovascular disease), thereby providing confidence in the utility. In contrast to previous studies based on latent feature analysis, we uncovered previously unknown, or hidden, factors. Based on these results, Bayesian nonparametric models may be useful for developing our understanding of complex comorbidity patterns and disease progression in chronic diseases. A deeper understanding of T2DM progression and multimorbidity can foster new hypotheses for further epidemiological studies and be used in clinical guidance of the patients.

## Methods

### Data source

The IQVIA Medical Research Database UK (IMRD-UK) incorporates data supplied from The Health Improvement Network (THIN), which is a Cegedim database of anonymized electronic health records generated from the daily record of General Practitioners (GPs). It includes data from more than 18 million patients from over 800 GP practices in the UK and about 6% of the UK population. The database contains detailed information about patient characteristics (i.e., year of birth, sex, practice registration date, practice de-registration date, ethnicity), medical conditions (i.e., diagnoses with dates, referrals to hospitals, symptoms), medications (i.e., drug name, formulation, date, strengths, quantity, dosing instructions), in practice immunizations, laboratory tests, and results, and other patient-level data (i.e., smoking status, height, weight, alcohol use, pregnancy, birth, death dates). For medical conditions, all diagnoses are coded according to the Read clinical code system, a comprehensive coding language with over 100,000 codes and are comparable to the international classification of diseases (ICD) system.

The IMRD contains routinely collected patient data from participating GP practices. Informed consent from all patients to have their data included in the IMRD is obtained by the GP and patients have the option to opt out of the data collection at any time. Ethical approval for the use of the IMRD for medical and public health research was approved by the London—South East Research Ethics Committee (Ref 18/LO/0441). Ethical approval for the protocol of this project was obtained by the IMRD Scientific Research Council (SRC reference number: 20SR062). All methods in this study were carried out in accordance with the Strengthening the Reporting of Observational Studies in Epidemiology (STROBE) reporting guideline and was performed according to the Declaration of Helsinki.

### Study population

To identify patients with T2DM, we included all adult patients (age 18 +) with a first-ever prescription of a non-insulin antidiabetic drug (NIAD) between January 1st 2006 and December 31st 2019. The date of the first NIAD prescription defined the index date (start of follow-up). In order to identify new users, patients were required to have a minimum of one year of valid data collection prior to the first-ever prescription of a NIAD. Patients with a history of polycystic ovarian syndrome (PCOS), gestational diabetes, or insulin prescription prior to the index date were excluded since these conditions are treated with NIAD, although not necessarily T2DM patients.

### Chronic disease conditions

Chronic diseases were identified as conditions that last longer than one year and require medical attention^24^. We selected 58 distinct chronic comorbidities using Read Codes Supplementary Table S1, the clinical terminology used in General Practice in the UK in which each Read Code represents a term or short phrase which describes a health-related concept^25^. Read Codes were simplified to the third level, i.e., the first three letters of the Read Code, to encompass all the possible and small deviations from the primary diagnosis. For example, a “conductive hearing loss”, with Read Code F590500, can be collapsed to “conductive deafness”, F590.11, or further summarised to “hearing loss”, F59.00. The selected chronic conditions were based on conditions from the Quality Outcome Framework (QOF) and previous studies on comorbidities commonly associated with T2DM^11,26,27^. Given the considerable heterogeneity in the pathogenesis and pathophysiology of cancer, we grouped all diagnoses of neoplasms under one category (Read Codes starting with B). We identified existing comorbidities if a patient had ever had a recorded diagnosis on or before the index date. Finally, to avoid convergence problems of the models, we selected those chronic comorbidities with a prevalence higher than 1.0% for males and females.

We created a longitudinal patient-disease binary matrix in discrete diabetes years (i.e., years elapsed between chronic disease onset and index date). Therefore, every row corresponded to a specific patient in a given year, and the columns corresponded to the comorbidities that the patient had developed in that time point. For model fitting, we selected the last observed period for each patient. Thus, we ended up with a single row per patient which encoded the chronic comorbidities that the patient had developed.

### Statistical methods

Prior to model development, we summarized main patient characteristics at index date, stratified by sex. Latent feature models assume that there is an unknown low-dimensional representation of patients-disease^28^. Traditional methods are matrix factorization or latent Dirichlet allocation (LDA)^29^. However, these approaches require that the number of latent features to be retrieved be specified and assumed to follow a specific distribution, e.g., Gaussian distribution. An elegant solution to these issues is achieved by using Bayesian nonparametric models, such as a General Latent Feature Model (GLFM), by posing an Indian Buffet Process (IBP) as nonparametric prior over binary observation matrices^30^. This generated a binary matrix where columns represent a potentially unlimited number of features, while rows, representing patients, are finite. Therefore, GLFMs conduct latent feature analysis without pre-specifying the number of latent features. Each data point ${x}_{n}^{d}$ can be explained by a *K*-length binary vector ${\mathbf{z}}_{n}=\left[{\mathrm{z}}_{n1},\dots ,{\mathrm{z}}_{nK}\right]$ whose elements indicate whether a latent feature is active or not for the ${n}^{th}$ object, and a real-valued weighting vector ${\mathbf{B}}^{d}=\left[{b}_{1}^{d},\dots ,{b}_{K}^{d}\right]$ whose elements ${b}_{k}^{d}$ weight the influence of each latent feature in the ${d}^{th}$ attribute of $\mathbf{X}$. Therefore, the likelihood can be described as:

$$p\left(\mathbf{X}|\mathbf{Z},{\{\mathbf{B}}^{{\varvec{d}}}{\}}_{{\varvec{d}}=1}^{{\varvec{D}}}\right)=\prod_{{\varvec{d}}=1}^{{\varvec{D}}}\prod_{{\varvec{n}}=1}^{{\varvec{N}}}{\varvec{p}}\left({x}_{n}^{d}|{\mathbf{z}}_{{\varvec{n}}},{\mathbf{B}}^{{\varvec{d}}}\right).$$

The binary latent feature vectors ${\mathbf{z}}_{n}$ are gathered in a $N\times K$ matrix $\mathbf{Z}$ which follows an IBP prior with $\alpha$ as a concentration parameter, i.e., $\mathbf{Z}\sim IBP\left(\alpha \right),$ where $\alpha$ controls the a priori activation probability of new features. Therefore, larger values will result in a higher number of expected latent features as well as a larger number of active features per row. For further details see Valera et al.^19^. Moreover, we forced the first latent feature to be always active, acting as a bias term (i.e., all patients who do not have comorbidities or just one random comorbidity would only have the first latent feature), making this group to act as a baseline cluster.

On the ${\mathbf{B}}^{d}$ matrix we place a Gaussian prior, ${\mathbf{B}}^{d}\sim N\left(0,{\sigma }_{B}^{2}{\mathbf{I}}_{K}\right)$. In order to overcome the problems of not having a Gaussian-distributed observation matrix, we transform each data point ${x}_{n}^{d}$ into an auxiliary Gaussian variable ${y}_{n}^{d}$, also called *pseudo-observation*, by applying a transformation function ${f}_{d}\left(\cdot \right)$. The *pseudo-observation* is defined as

$$p\left({y}_{n}^{d}|{\mathbf{z}}_{n},{\mathbf{B}}^{d}\right)=N\left({y}_{n}^{d}|{\mathbf{z}}_{n}{\mathbf{B}}^{d},{\sigma }_{y}^{2}\right).$$

In the case of a binary observation matrix $\mathbf{X}$ each observation ${x}_{n}^{d}$ can only take two values ${x}_{n}^{d}\in \left\{0, 1\right\}$. Hence, we can map the real values to the positive real numbers by applying the following transformation

$$x_{n}^{d} = f_{d} \left( {y_{n}^{d} } \right) = \left\lfloor {f_{{{\text{R}}_{ + } }} \left( {y_{n}^{d} } \right)} \right\rfloor = \left\lfloor {\frac{{ {\text{log}} \left( {{\text{exp}}\left( {y_{n}^{d} } \right) + 1} \right)}}{\omega } + \mu } \right\rfloor ,$$

where $\omega$ and $\mu$ are scale and location hyper-parameters. Hence, the likelihood is defined as

$$p\left({x}_{n}^{d}|{\mathbf{z}}_{n},{\mathbf{B}}^{d}\right)=\Phi \left(\frac{{f}^{-1}\left({x}_{n}^{d}+1\right)-{\mathbf{z}}_{n}{\mathbf{B}}^{d}}{{\sigma }_{y}}\right)-\Phi \left(\frac{{f}^{-1}\left({x}_{n}^{d}\right)-{\mathbf{z}}_{n}{\mathbf{B}}^{d}}{{\sigma }_{y}}\right),$$

where ${f}_{{\mathfrak{R}}_{+}}^{-1}: {\mathfrak{R}}_{+}\to \mathfrak{R}$ is the inverse function of the transformation ${f}_{{\mathfrak{R}}_{+}}\left(\cdot \right).$

### Inference

Given that the posterior distribution of ${\mathbf{B}}^{d}$ is intractable, we rely on a Markov Chain Monte Carlo (MCMC) approach, i.e., Gibbs sampling^19^, to obtain posterior samples from **Z** and **B**. In order to speed up the sampling process, those patients who did not have any comorbidity were not sampled, and were assigned only the bias term. The sampling procedure can be summarized as follows:

Firstly, we sample $\mathbf{Z}$

$$p\left({Z}_{nk}=1|{\mathbf{Z}}_{-nk},\mathbf{X}\right)\propto \frac{{\mathbf{m}}_{k}-{Z}_{nk}}{\mathrm{N}}p\left(\mathbf{X}|\mathbf{Z}\right),$$

then we sample ${\mathbf{B}}^{{\varvec{d}}}$

$$p\left({\mathbf{b}}^{d}|{\mathbf{y}}_{n}^{d},\mathbf{Z}\right)=N\left({\mathbf{b}}^{d}|{\mathbf{P}}^{-1}{{\varvec{\uplambda}}}^{d},{\mathbf{P}}^{-1}\right),$$

where $\mathbf{P}={\mathbf{Z}}^{\mathrm{\top }}\mathbf{Z}+1/{\upsigma }_{B}^{2}{\mathbf{I}}_{k}$ and ${{\varvec{\uplambda}}}^{d}={\mathbf{Z}}^{\mathrm{\top }}{\mathbf{y}}^{d}$. Finally, we sample ${Y}^{d}$ given $\mathbf{X},\mathbf{Z},{\mathbf{B}}^{\mathrm{d}}$,

$$p\left({y}_{n1}^{d}|{x}_{n}^{d},{\mathrm{z}}_{n},{\mathrm{B}}^{d}\right)=N\left({y}_{n1}^{d} |{\mathbf{z}}_{n}{\mathbf{b}}_{1}^{d},{\sigma }_{y}^{2}\right){\mathbb{I}}\left({f}_{{\mathfrak{R}}_{+}}^{-1}\left({x}_{n}^{d}\right)\le {y}_{n1}^{d}<{f}^{-1}\left({x}_{n}^{d}+1\right)\right),$$

where we sample ${y}_{n1}^{d}$ from a Gaussian left-truncated by ${f}_{{\mathfrak{R}}_{+}}^{-1}\left({x}_{n}^{d}\right)$ and right-truncated by ${f}_{{\mathfrak{R}}_{+}}^{-1}\left({x}_{n}^{d}+1\right)$. This inference procedure is repeated as many times as iterations set.

We set the Gibbs sampler to run for 1000 iterations, the variance of the Gaussian prior to the weighing vectors ${\mathbf{B}}^{\mathbf{d}}$ to ${\upsigma }_{B}^{2}=1$, and the concentration parameter for the IBP to $\mathrm{\alpha }=1$. In order to speed up the computations, we did not sample those rows of **Z** corresponding to patients with no disease.

### Predictions

In order to analyze the evolution of comorbidities over time, we estimated the active latent features in each period per patient. To do so, we retrieve all the unique combinations of latent features ${\mathbf{z}}_{i}$ from $\mathbf{Z}$ and compute the likelihood of each ${\mathbf{z}}_{i}$ to each observation ${x}_{n}$, as previously shown,

$$p\left({\mathbf{x}}_{{\varvec{n}}}|{\mathbf{z}}_{{\varvec{i}}},{\{\mathbf{B}}^{{\varvec{d}}}{\}}_{{\varvec{d}}=1}^{{\varvec{D}}}\right)=\prod_{{\varvec{d}}=1}^{{\varvec{D}}}p\left({x}_{n}^{d}|{\mathbf{z}}_{n},{\mathbf{B}}^{d}\right).$$

### Description of clusters

We described each cluster ${\mathbf{z}}_{i}$ and tabulated the count and proportion of patients with a specific disease within that cluster, the proportion of people with that specific disease in the overall population, and the Observed-Expected (O/E) ratio. The O/E ratio is the ratio between the proportion of patients with a given disease in a cluster divided by the proportion of patients with that disease overall, and it gives a magnitude of how a specific comorbidity is over- or underrepresented in a given cluster. Moreover, we reported the proportion of females within that cluster and in the population overall and computed the corresponding O/E ratio, the proportion of females in a cluster divided by the proportion of females overall, to detect if there were female-dominated clusters.

We reported the empirical probabilities of possessing at least one latent feature or a single feature. Additionally, we computed the empirical and the product probability of possessing at least two latent features to identify if two given latent features were independent. For instance, once a latent feature is active, the probability of having another given latent feature is higher. Finally, we also computed the probability of possessing at least latent features ${k}_{1}$ and ${k}_{2}$ given that ${k}_{1}$ is active, i.e.,

$$p\left({k}_{1}=1,{k}_{2}=1|{k}_{1}=1\right)\frac{{\sum }_{n=1}^{N}{z}_{n{k}_{1}}{z}_{n{k}_{2}}}{{\sum }_{n=1}^{N}{z}_{n{k}_{1}}}.$$

The Bayesian nonparametric model was implemented in C ++ , and all statistical analyses and summary statistics were done in R version 3.5.1 (R Project for Statistical Computing). Network visualization was done in Gephi^31^.

### Network visualization

To visualize the progression between clusters ${\mathbf{z}}_{i}$ over time we performed a network visualization. The history of latent membership for patient $n$ in time $t$ is represented by ${\mathbf{Z}}_{nt}$. Nodes represent the different clusters ${\mathbf{z}}_{i}$, and the directed edges the direction of the transition between clusters ${\mathbf{z}}_{i}$ in different times $t$. The size of the nodes, the weight, is proportional to the number of times patients were in that specific node. In order to improve the visualization of the nodes, we took the log of the node weight and rescaled the weights between 0 and 1 as follows:

$${w}_{i}=\frac{log\left({w}_{i}\right)-min\left(log\left(w\right)\right)}{max\left(log\left(w\right)\right)-min\left(log\left(w\right)\right)}.$$

### Ethical approval

Ethical approval for the protocol of this project was obtained by the THIN scientific research council (reference number: 20SR062).

## Supplementary Information

Supplementary Information.

## Data Availability

The data that support the findings of this study are available from IQVIA Medical Research Data (IMRD), a Cegedim Database, but restrictions apply to the availability of these data. The datasets generated and/or analyzed during the current study are not publicly available due to the fact that they were licensed for the sole use of the current study so they are not publicly available but are available from the corresponding author on reasonable request and with permission of IQVIA. For further information on how to access the data contact IQVIA at IMRDEnquiries@iqvia.com.

## References

[1] Papatheodorou K, Banach M, Bekiari E, Rizzo M, Edmonds M (2018). Complications of diabetes 2017. J. Diabetes Res..

[2] Iglay K (2016). Prevalence and co-prevalence of comorbidities among patients with type 2 diabetes mellitus. Curr. Med. Res. Opin..

[3] Adriaanse MC, Drewes HW, van der Heide I, Struijs JN, Baan CA (2016). The impact of comorbid chronic conditions on quality of life in type 2 diabetes patients. Qual. Life Res..

[4] Lipska KJ, Krumholz H, Soones T, Lee SJ (2016). Polypharmacy in the aging patient: A review of glycemic control in older adults with type 2 diabetes. JAMA.

[5] Aguado A, Moratalla-Navarro F, López-Simarro F, Moreno V (2020). MorbiNet: Multimorbidity networks in adult general population. Analysis of type 2 diabetes mellitus comorbidity. Sci. Rep..

[6] Khan A, Uddin S, Srinivasan U (2018). Comorbidity network for chronic disease: A novel approach to understand type 2 diabetes progression. Int. J. Med. Inform..

[7] Ahlqvist E (2018). Novel subgroups of adult-onset diabetes and their association with outcomes: A data-driven cluster analysis of six variables. Lancet Diabetes Endocrinol..

[8] Dennis JM, Shields BM, Henley WE, Jones AG, Hattersley AT (2019). Disease progression and treatment response in data-driven subgroups of type 2 diabetes compared with models based on simple clinical features: An analysis using clinical trial data. Lancet Diabetes Endocrinol..

[9] Islam MM (2014). Multimorbidity and comorbidity of chronic diseases among the senior Australians: Prevalence and patterns. PLoS ONE.

[10] Cornell JE (2008). Multimorbidity clusters: Clustering binary data from multimorbidity clusters: Clustering binary data from a large administrative medical database. Appl. Multivar. Res..

[11] Strauss VY, Jones PW, Kadam UT, Jordan KP (2014). Distinct trajectories of multimorbidity in primary care were identified using latent class growth analysis. J. Clin. Epidemiol..

[12] Ferguson TS (1973). A Bayesian analysis of some nonparametric problems. Ann. Stat.

[13] Antoniak CE (1974). Mixtures of dirichlet processes with applications to Bayesian nonparametric problems. Ann. Stat..

[14] Ruiz FJR, Valera I, Blanco C, Perez-Cruz FO (2014). Bayesian nonparametric comorbidity analysis of psychiatric disorders. J. Mach. Learn Res..

[15] Ruiz, F., Valera, I., Blanco, C. & Pérez-Cruz, F. Bayesian Nonparametric Modeling of Suicide Attempts. *Adv Neural Inf Process Syst.* **25**, 1853–1861 (2012).

[16] Dagliati A (2018). Machine learning methods to predict diabetes complications. J. Diabetes Sci. Technol..

[17] Cahn A (2020). Prediction of progression from pre-diabetes to diabetes: Development and validation of a machine learning model. Diabetes Metab. Res. Rev..

[18] Oh W (2016). Type 2 diabetes mellitus trajectories and associated risks. Big Data.

[19] Valera I, Pradier MF, Lomeli M, Ghahramani Z (2020). General latent feature models for heterogeneous datasets. J. Mach. Learn Res..

[20] Marrie RA (2021). The relationship between symptoms of depression and anxiety and disease activity in IBD over time. Inflamm. Bowel Dis..

[21] Bannaga AS, Selinger CP (2015). Inflammatory bowel disease and anxiety: Links, risks, and challenges faced. Clin. Exp. Gastroenterol..

[22] Siegmann E-M (2018). Association of depression and anxiety disorders with autoimmune thyroiditis: A systematic review and meta-analysis. JAMA Psychiat..

[23] Constant EL (2005). Anxiety and depression, attention and executive functions in hypothyroidism. J. Int. Neuropsychol. Soc..

[24] Bernell S, Howard SW (2016). Use your words carefully: What is a chronic disease?. Front. Public Health.

[25] Booth N (1994). What are the read codes?. Health Libr. Rev..

[26] Charlson ME, Pompei P, Ales KL, MacKenzie CR (1987). A new method of classifying prognostic comorbidity in longitudinal studies: Development and validation. J. Chron. Dis..

[27] Barnett K (2012). Epidemiology of multimorbidity and implications for health care, research, and medical education: A cross-sectional study. Lancet.

[28] Griffiths TL, Ghahramani Z (2011). The Indian buffet process: An introduction and review. J. Mach. Learn Res..

[29] Blei DM (2014). Build, compute, critique, repeat: Data analysis with latent variable models. Annu. Rev. Stat. Appl..

[30] Thibaux, R. & Jordan, M. I. Hierarchical Beta Processes and the Indian Buffet Process. in *International Conference on Artificial Intelligence and Statistics*, 564–571 (PMLR, 2007).

[31] Bastian M, Heymann S, Jacomy M (2009). Gephi: An open source software for exploring and manipulating networks. Proc. Int. AAAI Conf. Web Social Media.

