# Supplementary material for: Comorbidity clusters associated with newly treated type 2 diabetes mellitus: a Bayesian nonparametric analysis
Source: Sci Rep. 2022 Nov 30;12:20653. doi: 10.1038/s41598-022-24217-2 (PMC9712684; doi:10.1038/s41598-022-24217-2)
Supplement: Supplementary file 1 — Supplementary Information. [file 41598_2022_24217_MOESM1_ESM.pdf]

Comorbidity clusters associated with newly treated Type 2 diabetes mellitus: a Bayesian nonparametric analysis

Martinez-De la Torre A, Perez-Cruz F, Weiler S, Burden AM

Supplementary Tables and Figures

Tables

Supplementary Table S1. All chronic comorbidities selected for screening and whether they were included in the final analysis.

Supplementary Table S2. Description of clusters after Bayesian nonparametric model.

Supplementary Table S3. Comparison of disease prevalence between the three main cardiovascular clusters.

Supplementary Table S4. Demographic characteristics of 175,383 T2DM patients at index date (first NIAD prescription) stratified by cluster, first 20 clusters.

Figures

Supplementary Figure S1. O/E ratios for women for the first 20 clusters. The bars above O/E ratios 1.0 indicate that that specific cluster had a higher proportion of females than males. The colours represent the proportion of male and females in each cluster.

Supplementary Figure S2. Network representation of the transitions between first 20 clusters. Nodes represent clusters, edges indicate the transition between two given clusters, and the node size corresponds to the logarithm of the size (number of patients in that node at any time). The nodes of clusters 15 to 20 had a low proportion of patients, thus a very small node size.

Supplementary Table S2. All chronic comorbidities selected for screening and whether they were included in the final analysis. In order to avoid convergence problems only comorbidities with a prevalence higher than 1% in the last period both overall and stratified by sex were included.

Read Code	Description	Included	Count (%)
B	Cancer	Yes	16,981 (9.7)
G20	High blood pressure	Yes	11,182 (6.4)
N05	Osteoarthritis	Yes	9210 (5.3)
F46	Senile cataract	Yes	7890 (4.5)
G57	Atrial fibrillation	Yes	7167 (4.1)
C32	Pure hypercholesterolaemia	Yes	6221 (3.5)
F59	Deafness	Yes	5442 (3.1)
J52	Irritable bowel syndrome	Yes	5517 (3.1)
E20	Anxiety & other neurotic, stress related & somatoform disorders	Yes	4830 (2.8)
J61	Chronic liver disease	Yes	4734 (2.7)
G58	Congestive heart failure	Yes	4058 (2.3)
C04	Hypothyroidism	Yes	3545 (2)
C38	Obesity	Yes	3283 (1.9)
N33	Osteoporosis	Yes	3381 (1.9)
G73	Intermittent claudication	Yes	2766 (1.6)
F45	Primary open-angle glaucoma	Yes	2410 (1.4)
H31	Chronic bronchitis	Yes	2297 (1.3)
N06	Arthropathy	Yes	2245 (1.3)
F36	Hereditary and idiopathic peripheral neuropathy	Yes	2038 (1.2)
M16	Psoriasis or eczema	Yes	2156 (1.2)
K05	Chronic kidney disease	Yes	1902 (1.1)
N11	Cervical spondylosis	Yes	2011 (1.1)
G33	Angina pectoris	Yes	1825 (1)
K20	Prostatism	No	1893 (1.1)
G34	Ischaemic heart disease	No	1161 (0.7)
E00	Senile/presenile dementia	No	684 (0.4)
H34	Bronchiectasis	No	651 (0.4)
N04	Rheumatoid arthritis	No	789 (0.4)
F12	Parkinson’s disease	No	590 (0.3)
F49	Blindness & low vision	No	532 (0.3)
H56	Diffuse pulmonary fibrosis	No	450 (0.3)
F25	Epilepsy	No	413 (0.2)
G67	Cerebral atherosclerosis	No	321 (0.2)
H32	Emphysema	No	371 (0.2)
H54	Pulmonary oedema	No	391 (0.2)
J41	Ulcerative Colitis	No	286 (0.2)
K11	Hydronephrosis	No	384 (0.2)
A70	Viral Hepatitis	No	100 (0.1)
D10	Hereditary haemolytic anaemia	No	155 (0.1)
E10	Schizophrenia (and related non-organic psychosis) or bipolar disorder	No	113 (0.1)
E2B1	Chronic Depression	No	160 (0.1)
F22	Hemiplegia or paraplegia	No	218 (0.1)
F24	Hemiplegia or paraplegia	No	205 (0.1)
G11	Mitral stenosis	No	97 (0.1)
J40	Crohn's Disease	No	136 (0.1)
F20	Multiple sclerosis	No	74 (0)
G21	Hypertensive heart disease	No	45 (0)
G22	Hypertensive renal disease	No	24 (0)
H35	Extrinsic allergic alveolitis	No	54 (0)
J13	Peptic ulcer disease	No	30 (0)
K03	Nephritis and nephropathy	No	31 (0)
N31	Paget's disease of bone	No	62 (0)
SC2	Late effect - nervous system injury	No	39 (0)

Supplementary Table S2. Description of clusters after Bayesian nonparametric model.

Cluster	Latent features	Read term	Count	N cluster	Count prop	Total disease	Total dis. Prop	O/E ratio
1	LF1	High blood pressure	8396	147,816	5.7%	10,660	6.1%	0.93
1	LF1	Pure hypercholesterolaemia	4570	147,816	3.1%	5938	3.4%	0.91
1	LF1	Chronic liver disease	3437	147,816	2.3%	4505	2.6%	0.91
1	LF1	Anxiety & other	3101	147,816	2.1%	4115	2.3%	0.89
1	LF1	Cancer	11,985	147,816	8.1%	15,937	9.1%	0.89
1	LF1	Irritable bowel syndrome	3580	147,816	2.4%	5019	2.9%	0.85
1	LF1	Deafness	3721	147,816	2.5%	5261	3.0%	0.84
1	LF1	Osteoarthritis	6292	147,816	4.3%	8954	5.1%	0.83
1	LF1	Senile cataract	5139	147,816	3.5%	7709	4.4%	0.79
1	LF1	Atrial fibrillation	4299	147,816	2.9%	7000	4.0%	0.73
1	LF1	Psoriasis or eczema	0	147,816	0.0%	1975	1.1%	0.00
1	LF1	Arthropathy	0	147,816	0.0%	2168	1.2%	0.00
1	LF1	Obesity	0	147,816	0.0%	2879	1.6%	0.00
1	LF1	Cervical spondylosis	0	147,816	0.0%	1937	1.1%	0.00
1	LF1	Primary open-angle glaucoma	0	147,816	0.0%	2330	1.3%	0.00
1	LF1	Chronic kidney disease	0	147,816	0.0%	1869	1.1%	0.00
1	LF1	Chronic bronchitis	0	147,816	0.0%	2260	1.3%	0.00
1	LF1	Angina pectoris	0	147,816	0.0%	1773	1.0%	0.00
1	LF1	Congestive heart failure	0	147,816	0.0%	3962	2.3%	0.00
1	LF1	Intermittent claudication	0	147,816	0.0%	2653	1.5%	0.00
1	LF1	Hypothyroidism	0	147,816	0.0%	3260	1.9%	0.00
1	LF1	Neuropathy	0	147,816	0.0%	1964	1.1%	0.00
1	LF1	Osteoporosis	0	147,816	0.0%	3233	1.8%	0.00

Definitions: Count, numbers of patients with that disease in that cluster; N cluster, total number of individuals within that cluster; Count prop, proportion of patients who have that disease within a cluster; Total disease, overall number of patients with that disease; Total dis. Prop, overall proportion that have that disease; O/E ratio, observed to expected ratio.

Supplementary Table S2 (continued). Description of clusters after Bayesian nonparametric model.

Cluster	Latent features	Read term	Count	N cluster	Count prop	Total disease	Total dis. Prop	O/E ratio
2	LF1 LF2	Congestive heart failure	2711	2766	98.0%	3962	2.3%	43.39
2	LF1 LF2	Atrial fibrillation	776	2766	28.1%	7000	4.0%	7.03
2	LF1 LF2	Senile cataract	281	2766	10.2%	7709	4.4%	2.31
2	LF1 LF2	Deafness	168	2766	6.1%	5261	3.0%	2.02
2	LF1 LF2	Irritable bowel syndrome	141	2766	5.1%	5019	2.9%	1.78
2	LF1 LF2	Cancer	395	2766	14.3%	15,937	9.1%	1.57
2	LF1 LF2	Osteoarthritis	183	2766	6.6%	8954	5.1%	1.30
2	LF1 LF2	Anxiety & other	75	2766	2.7%	4115	2.3%	1.16
2	LF1 LF2	Chronic liver disease	82	2766	3.0%	4505	2.6%	1.15
2	LF1 LF2	High blood pressure	151	2766	5.5%	10,660	6.1%	0.90
2	LF1 LF2	Pure hypercholesterolaemia	77	2766	2.8%	5938	3.4%	0.82
2	LF1 LF2	Osteoporosis	0	2766	0.0%	3233	1.8%	0.00
2	LF1 LF2	Obesity	0	2766	0.0%	2879	1.6%	0.00
2	LF1 LF2	Cervical spondylosis	0	2766	0.0%	1937	1.1%	0.00
2	LF1 LF2	Angina pectoris	0	2766	0.0%	1773	1.0%	0.00
2	LF1 LF2	Arthropathy	0	2766	0.0%	2168	1.2%	0.00
2	LF1 LF2	Intermittent claudication	0	2766	0.0%	2653	1.5%	0.00
2	LF1 LF2	Psoriasis or eczema	0	2766	0.0%	1975	1.1%	0.00
2	LF1 LF2	Neuropathy	0	2766	0.0%	1964	1.1%	0.00
2	LF1 LF2	Hypothyroidism	0	2766	0.0%	3260	1.9%	0.00
2	LF1 LF2	Chronic kidney disease	0	2766	0.0%	1869	1.1%	0.00
2	LF1 LF2	Primary open-angle glaucoma	0	2766	0.0%	2330	1.3%	0.00
2	LF1 LF2	Chronic bronchitis	0	2766	0.0%	2260	1.3%	0.00

Definitions: Count, numbers of patients with that disease in that cluster; N cluster, total number of individuals within that cluster; Count prop, proportion of patients who have that disease within a cluster; Total disease, overall number of patients with that disease; Total dis. Prop, overall proportion that have that disease; O/E ratio, observed to expected ratio

Supplementary Table S2. (continued) Description of clusters after Bayesian nonparametric model.

Cluster	Latent features	Read term	Count	N cluster	Count prop	Total disease	Total dis. Prop	O/E ratio
3	LF1 LF3	Hypothyroidism	2535	2572	98.6%	3260	1.9%	53.02
3	LF1 LF3	Irritable bowel syndrome	118	2572	4.6%	5019	2.9%	1.60
3	LF1 LF3	Anxiety & other	92	2572	3.6%	4115	2.3%	1.52
3	LF1 LF3	Senile cataract	168	2572	6.5%	7709	4.4%	1.49
3	LF1 LF3	Pure hypercholesterolaemia	129	2572	5.0%	5938	3.4%	1.48
3	LF1 LF3	Cancer	342	2572	13.3%	15,937	9.1%	1.46
3	LF1 LF3	Chronic liver disease	95	2572	3.7%	4505	2.6%	1.44
3	LF1 LF3	Osteoarthritis	184	2572	7.2%	8954	5.1%	1.40
3	LF1 LF3	High blood pressure	213	2572	8.3%	10,660	6.1%	1.36
3	LF1 LF3	Deafness	98	2572	3.8%	5261	3.0%	1.27
3	LF1 LF3	Atrial fibrillation	121	2572	4.7%	7000	4.0%	1.18
3	LF1 LF3	Congestive heart failure	0	2572	0.0%	3962	2.3%	0.00
3	LF1 LF3	Neuropathy	0	2572	0.0%	1964	1.1%	0.00
3	LF1 LF3	Obesity	0	2572	0.0%	2879	1.6%	0.00
3	LF1 LF3	Primary open-angle glaucoma	0	2572	0.0%	2330	1.3%	0.00
3	LF1 LF3	Angina pectoris	0	2572	0.0%	1773	1.0%	0.00
3	LF1 LF3	Psoriasis or eczema	0	2572	0.0%	1975	1.1%	0.00
3	LF1 LF3	Arthropathy	0	2572	0.0%	2168	1.2%	0.00
3	LF1 LF3	Osteoporosis	0	2572	0.0%	3233	1.8%	0.00
3	LF1 LF3	Chronic kidney disease	0	2572	0.0%	1869	1.1%	0.00
3	LF1 LF3	Cervical spondylosis	0	2572	0.0%	1937	1.1%	0.00
3	LF1 LF3	Intermittent claudication	0	2572	0.0%	2653	1.5%	0.00
3	LF1 LF3	Chronic bronchitis	0	2572	0.0%	2260	1.3%	0.00

Definitions: Count, numbers of patients with that disease in that cluster; N cluster, total number of individuals within that cluster; Count prop, proportion of patients who have that disease within a cluster; Total disease, overall number of patients with that disease; Total dis. Prop, overall proportion that have that disease; O/E ratio, observed to expected ratio.

Supplementary Table S2. (continued) Description of clusters after Bayesian nonparametric model.

Cluster	Latent features	Read term	Count	N cluster	Count prop	Total disease	Total dis. Prop	O/E ratio
4	LF1 LF4	Osteoporosis	2323	2350	98.9%	3233	1.8%	53.62
4	LF1 LF4	Senile cataract	269	2350	11.4%	7709	4.4%	2.60
4	LF1 LF4	Irritable bowel syndrome	148	2350	6.3%	5019	2.9%	2.20
4	LF1 LF4	Deafness	144	2350	6.1%	5261	3.0%	2.04
4	LF1 LF4	Osteoarthritis	212	2350	9.0%	8954	5.1%	1.77
4	LF1 LF4	Atrial fibrillation	162	2350	6.9%	7000	4.0%	1.73
4	LF1 LF4	Cancer	347	2350	14.8%	15,937	9.1%	1.62
4	LF1 LF4	Anxiety & other	81	2350	3.4%	4115	2.3%	1.47
4	LF1 LF4	Chronic liver disease	85	2350	3.6%	4505	2.6%	1.41
4	LF1 LF4	Pure hypercholesterolaemia	90	2350	3.8%	5938	3.4%	1.13
4	LF1 LF4	High blood pressure	157	2350	6.7%	10,660	6.1%	1.10
4	LF1 LF4	Hypothyroidism	0	2350	0.0%	3260	1.9%	0.00
4	LF1 LF4	Obesity	0	2350	0.0%	2879	1.6%	0.00
4	LF1 LF4	Congestive heart failure	0	2350	0.0%	3962	2.3%	0.00
4	LF1 LF4	Angina pectoris	0	2350	0.0%	1773	1.0%	0.00
4	LF1 LF4	Intermittent claudication	0	2350	0.0%	2653	1.5%	0.00
4	LF1 LF4	Neuropathy	0	2350	0.0%	1964	1.1%	0.00
4	LF1 LF4	Chronic kidney disease	0	2350	0.0%	1869	1.1%	0.00
4	LF1 LF4	Cervical spondylosis	0	2350	0.0%	1937	1.1%	0.00
4	LF1 LF4	Arthropathy	0	2350	0.0%	2168	1.2%	0.00
4	LF1 LF4	Psoriasis or eczema	0	2350	0.0%	1975	1.1%	0.00
4	LF1 LF4	Chronic bronchitis	0	2350	0.0%	2260	1.3%	0.00
4	LF1 LF4	Primary open-angle glaucoma	0	2350	0.0%	2330	1.3%	0.00

Definitions: Count, numbers of patients with that disease in that cluster; N cluster, total number of individuals within that cluster; Count prop, proportion of patients who have that disease within a cluster; Total disease, overall number of patients with that disease; Total dis. Prop, overall proportion that have that disease; O/E ratio, observed to expected ratio.

Supplementary Table S2. (continued) Description of clusters after Bayesian nonparametric model.

Cluster	Latent features	Read term	Count	N cluster	Count prop	Total disease	Total dis. Prop	O/E ratio
5	LF1 LF5	Obesity	2235	2252	99.2%	2879	1.6%	60.46
5	LF1 LF5	Pure hypercholesterolaemia	185	2252	8.2%	5938	3.4%	2.43
5	LF1 LF5	Chronic liver disease	121	2252	5.4%	4505	2.6%	2.09
5	LF1 LF5	High blood pressure	276	2252	12.3%	10,660	6.1%	2.02
5	LF1 LF5	Anxiety & other	105	2252	4.7%	4115	2.3%	1.99
5	LF1 LF5	Irritable bowel syndrome	101	2252	4.5%	5019	2.9%	1.57
5	LF1 LF5	Osteoarthritis	179	2252	7.9%	8954	5.1%	1.56
5	LF1 LF5	Cancer	262	2252	11.6%	15,937	9.1%	1.28
5	LF1 LF5	Atrial fibrillation	112	2252	5.0%	7000	4.0%	1.25
5	LF1 LF5	Deafness	63	2252	2.8%	5261	3.0%	0.93
5	LF1 LF5	Senile cataract	89	2252	4.0%	7709	4.4%	0.90
5	LF1 LF5	Hypothyroidism	0	2252	0.0%	3260	1.9%	0.00
5	LF1 LF5	Psoriasis or eczema	0	2252	0.0%	1975	1.1%	0.00
5	LF1 LF5	Neuropathy	0	2252	0.0%	1964	1.1%	0.00
5	LF1 LF5	Angina pectoris	0	2252	0.0%	1773	1.0%	0.00
5	LF1 LF5	Chronic kidney disease	0	2252	0.0%	1869	1.1%	0.00
5	LF1 LF5	Arthropathy	0	2252	0.0%	2168	1.2%	0.00
5	LF1 LF5	Chronic bronchitis	0	2252	0.0%	2260	1.3%	0.00
5	LF1 LF5	Cervical spondylosis	0	2252	0.0%	1937	1.1%	0.00
5	LF1 LF5	Primary open-angle glaucoma	0	2252	0.0%	2330	1.3%	0.00
5	LF1 LF5	Intermittent claudication	0	2252	0.0%	2653	1.5%	0.00
5	LF1 LF5	Osteoporosis	0	2252	0.0%	3233	1.8%	0.00
5	LF1 LF5	Congestive heart failure	0	2252	0.0%	3962	2.3%	0.00

Definitions: Count, numbers of patients with that disease in that cluster; N cluster, total number of individuals within that cluster; Count prop, proportion of patients who have that disease within a cluster; Total disease, overall number of patients with that disease; Total dis. Prop, overall proportion that have that disease; O/E ratio, observed to expected ratio.

Supplementary Table S2. (continued) Description of clusters after Bayesian nonparametric model.

Cluster	Latent features	Read term	Count	N cluster	Count prop	Total disease	Total dis. Prop	O/E ratio
6	LF1 LF6	Intermittent claudication	1855	1871	99.1%	2653	1.5%	65.54
6	LF1 LF6	Atrial fibrillation	153	1871	8.2%	7000	4.0%	2.05
6	LF1 LF6	Senile cataract	153	1871	8.2%	7709	4.4%	1.86
6	LF1 LF6	Deafness	96	1871	5.1%	5261	3.0%	1.71
6	LF1 LF6	Irritable bowel syndrome	84	1871	4.5%	5019	2.9%	1.57
6	LF1 LF6	Cancer	260	1871	13.9%	15,937	9.1%	1.53
6	LF1 LF6	High blood pressure	159	1871	8.5%	10,660	6.1%	1.40
6	LF1 LF6	Osteoarthritis	129	1871	6.9%	8954	5.1%	1.35
6	LF1 LF6	Pure hypercholesterolaemia	78	1871	4.2%	5938	3.4%	1.23
6	LF1 LF6	Anxiety & other	49	1871	2.6%	4115	2.3%	1.12
6	LF1 LF6	Chronic liver disease	33	1871	1.8%	4505	2.6%	0.69
6	LF1 LF6	Osteoporosis	0	1871	0.0%	3233	1.8%	0.00
6	LF1 LF6	Hypothyroidism	0	1871	0.0%	3260	1.9%	0.00
6	LF1 LF6	Congestive heart failure	0	1871	0.0%	3962	2.3%	0.00
6	LF1 LF6	Arthropathy	0	1871	0.0%	2168	1.2%	0.00
6	LF1 LF6	Primary open-angle glaucoma	0	1871	0.0%	2330	1.3%	0.00
6	LF1 LF6	Angina pectoris	0	1871	0.0%	1773	1.0%	0.00
6	LF1 LF6	Cervical spondylosis	0	1871	0.0%	1937	1.1%	0.00
6	LF1 LF6	Psoriasis or eczema	0	1871	0.0%	1975	1.1%	0.00
6	LF1 LF6	Chronic bronchitis	0	1871	0.0%	2260	1.3%	0.00
6	LF1 LF6	Obesity	0	1871	0.0%	2879	1.6%	0.00
6	LF1 LF6	Chronic kidney disease	0	1871	0.0%	1869	1.1%	0.00
6	LF1 LF6	Neuropathy	0	1871	0.0%	1964	1.1%	0.00

Definitions: Count, numbers of patients with that disease in that cluster; N cluster, total number of individuals within that cluster; Count prop, proportion of patients who have that disease within a cluster; Total disease, overall number of patients with that disease; Total dis. Prop, overall proportion that have that disease; O/E ratio, observed to expected ratio.

Supplementary Table S2. (continued) Description of clusters after Bayesian nonparametric model.

Cluster	Latent features	Read term	Count	N cluster	Count prop	Total disease	Total dis. Prop	O/E ratio
7	LF1 LF7	Primary open-angle glaucoma	1789	1795	99.7%	2330	1.3%	75.02
7	LF1 LF7	Senile cataract	289	1795	16.1%	7709	4.4%	3.66
7	LF1 LF7	Osteoarthritis	143	1795	8.0%	8954	5.1%	1.56
7	LF1 LF7	Deafness	81	1795	4.5%	5261	3.0%	1.50
7	LF1 LF7	Cancer	234	1795	13.0%	15,937	9.1%	1.43
7	LF1 LF7	High blood pressure	144	1795	8.0%	10,660	6.1%	1.32
7	LF1 LF7	Pure hypercholesterolaemia	79	1795	4.4%	5938	3.4%	1.30
7	LF1 LF7	Anxiety & other	52	1795	2.9%	4115	2.3%	1.23
7	LF1 LF7	Chronic liver disease	55	1795	3.1%	4505	2.6%	1.19
7	LF1 LF7	Atrial fibrillation	84	1795	4.7%	7000	4.0%	1.17
7	LF1 LF7	Irritable bowel syndrome	60	1795	3.3%	5019	2.9%	1.17
7	LF1 LF7	Cervical spondylosis	0	1795	0.0%	1937	1.1%	0.00
7	LF1 LF7	Arthropathy	0	1795	0.0%	2168	1.2%	0.00
7	LF1 LF7	Psoriasis or eczema	0	1795	0.0%	1975	1.1%	0.00
7	LF1 LF7	Angina pectoris	0	1795	0.0%	1773	1.0%	0.00
7	LF1 LF7	Chronic bronchitis	0	1795	0.0%	2260	1.3%	0.00
7	LF1 LF7	Osteoporosis	0	1795	0.0%	3233	1.8%	0.00
7	LF1 LF7	Hypothyroidism	0	1795	0.0%	3260	1.9%	0.00
7	LF1 LF7	Congestive heart failure	0	1795	0.0%	3962	2.3%	0.00
7	LF1 LF7	Intermittent claudication	0	1795	0.0%	2653	1.5%	0.00
7	LF1 LF7	Obesity	0	1795	0.0%	2879	1.6%	0.00
7	LF1 LF7	Chronic kidney disease	0	1795	0.0%	1869	1.1%	0.00
7	LF1 LF7	Neuropathy	0	1795	0.0%	1964	1.1%	0.00

Definitions: Count, numbers of patients with that disease in that cluster; N cluster, total number of individuals within that cluster; Count prop, proportion of patients who have that disease within a cluster; Total disease, overall number of patients with that disease; Total dis. Prop, overall proportion that have that disease; O/E ratio, observed to expected ratio.

Supplementary Table S2. (continued) Description of clusters after Bayesian nonparametric model.

Cluster	Latent features	Read term	Count	N cluster	Count prop	Total disease	Total dis. Prop	O/E ratio
8	LF1 LF9	Arthropathy	1531	1532	99.9%	2168	1.2%	80.84
8	LF1 LF9	Osteoarthritis	290	1532	18.9%	8954	5.1%	3.71
8	LF1 LF9	Anxiety & other	70	1532	4.6%	4115	2.3%	1.95
8	LF1 LF9	Deafness	82	1532	5.4%	5261	3.0%	1.78
8	LF1 LF9	Irritable bowel syndrome	74	1532	4.8%	5019	2.9%	1.69
8	LF1 LF9	Cancer	206	1532	13.4%	15,937	9.1%	1.48
8	LF1 LF9	Senile cataract	97	1532	6.3%	7709	4.4%	1.44
8	LF1 LF9	Pure hypercholesterolaemia	73	1532	4.8%	5938	3.4%	1.41
8	LF1 LF9	High blood pressure	129	1532	8.4%	10,660	6.1%	1.39
8	LF1 LF9	Chronic liver disease	54	1532	3.5%	4505	2.6%	1.37
8	LF1 LF9	Atrial fibrillation	73	1532	4.8%	7000	4.0%	1.19
8	LF1 LF9	Cervical spondylosis	0	1532	0.0%	1937	1.1%	0.00
8	LF1 LF9	Osteoporosis	0	1532	0.0%	3233	1.8%	0.00
8	LF1 LF9	Hypothyroidism	0	1532	0.0%	3260	1.9%	0.00
8	LF1 LF9	Chronic kidney disease	0	1532	0.0%	1869	1.1%	0.00
8	LF1 LF9	Chronic bronchitis	0	1532	0.0%	2260	1.3%	0.00
8	LF1 LF9	Psoriasis or eczema	0	1532	0.0%	1975	1.1%	0.00
8	LF1 LF9	Angina pectoris	0	1532	0.0%	1773	1.0%	0.00
8	LF1 LF9	Intermittent claudication	0	1532	0.0%	2653	1.5%	0.00
8	LF1 LF9	Congestive heart failure	0	1532	0.0%	3962	2.3%	0.00
8	LF1 LF9	Neuropathy	0	1532	0.0%	1964	1.1%	0.00
8	LF1 LF9	Obesity	0	1532	0.0%	2879	1.6%	0.00
8	LF1 LF9	Primary open-angle glaucoma	0	1532	0.0%	2330	1.3%	0.00

Definitions: Count, numbers of patients with that disease in that cluster; N cluster, total number of individuals within that cluster; Count prop, proportion of patients who have that disease within a cluster; Total disease, overall number of patients with that disease; Total dis. Prop, overall proportion that have that disease; O/E ratio, observed to expected ratio.

Supplementary Table S2. (continued) Description of clusters after Bayesian nonparametric model.

Cluster	Latent features	Read term	Count	N cluster	Count prop	Total disease	Total dis. Prop	O/E ratio
9	LF1 LF8	Chronic bronchitis	1494	1496	99.9%	2260	1.3%	77.50
9	LF1 LF8	Deafness	95	1496	6.4%	5261	3.0%	2.12
9	LF1 LF8	Senile cataract	134	1496	9.0%	7709	4.4%	2.04
9	LF1 LF8	Atrial fibrillation	121	1496	8.1%	7000	4.0%	2.03
9	LF1 LF8	Irritable bowel syndrome	85	1496	5.7%	5019	2.9%	1.99
9	LF1 LF8	Cancer	223	1496	14.9%	15,937	9.1%	1.64
9	LF1 LF8	Anxiety & other	52	1496	3.5%	4115	2.3%	1.48
9	LF1 LF8	Osteoarthritis	112	1496	7.5%	8954	5.1%	1.47
9	LF1 LF8	Chronic liver disease	38	1496	2.5%	4505	2.6%	0.99
9	LF1 LF8	Pure hypercholesterolaemia	47	1496	3.1%	5938	3.4%	0.93
9	LF1 LF8	High blood pressure	75	1496	5.0%	10,660	6.1%	0.82
9	LF1 LF8	Cervical spondylosis	0	1496	0.0%	1937	1.1%	0.00
9	LF1 LF8	Chronic kidney disease	0	1496	0.0%	1869	1.1%	0.00
9	LF1 LF8	Osteoporosis	0	1496	0.0%	3233	1.8%	0.00
9	LF1 LF8	Congestive heart failure	0	1496	0.0%	3962	2.3%	0.00
9	LF1 LF8	Arthropathy	0	1496	0.0%	2168	1.2%	0.00
9	LF1 LF8	Psoriasis or eczema	0	1496	0.0%	1975	1.1%	0.00
9	LF1 LF8	Angina pectoris	0	1496	0.0%	1773	1.0%	0.00
9	LF1 LF8	Primary open-angle glaucoma	0	1496	0.0%	2330	1.3%	0.00
9	LF1 LF8	Intermittent claudication	0	1496	0.0%	2653	1.5%	0.00
9	LF1 LF8	Neuropathy	0	1496	0.0%	1964	1.1%	0.00
9	LF1 LF8	Hypothyroidism	0	1496	0.0%	3260	1.9%	0.00
9	LF1 LF8	Obesity	0	1496	0.0%	2879	1.6%	0.00

Definitions: Count, numbers of patients with that disease in that cluster; N cluster, total number of individuals within that cluster; Count prop, proportion of patients who have that disease within a cluster; Total disease, overall number of patients with that disease; Total dis. Prop, overall proportion that have that disease; O/E ratio, observed to expected ratio.

Supplementary Table S2. (continued) Description of clusters after Bayesian nonparametric model.

**Cluster**	**Latent features**	**Read term**	**Count**	**N cluster**	**Count prop**	**Total disease**	**Total dis. Prop**	**O/E ratio**
10	LF1 LF10	Psoriasis or eczema	1477	1481	99.7%	1975	1.1%	88.56
10	LF1 LF10	Osteoarthritis	144	1481	9.7%	8954	5.1%	1.90
10	LF1 LF10	Chronic liver disease	71	1481	4.8%	4505	2.6%	1.87
10	LF1 LF10	Anxiety & other	56	1481	3.8%	4115	2.3%	1.61
10	LF1 LF10	High blood pressure	139	1481	9.4%	10,660	6.1%	1.54
10	LF1 LF10	Pure hypercholesterolaemia	77	1481	5.2%	5938	3.4%	1.54
10	LF1 LF10	Irritable bowel syndrome	63	1481	4.3%	5019	2.9%	1.49
10	LF1 LF10	Cancer	185	1481	12.5%	15,937	9.1%	1.37
10	LF1 LF10	Deafness	52	1481	3.5%	5261	3.0%	1.17
10	LF1 LF10	Senile cataract	75	1481	5.1%	7709	4.4%	1.15
10	LF1 LF10	Atrial fibrillation	57	1481	3.8%	7000	4.0%	0.96
10	LF1 LF10	Intermittent claudication	0	1481	0.0%	2653	1.5%	0.00
10	LF1 LF10	Obesity	0	1481	0.0%	2879	1.6%	0.00
10	LF1 LF10	Congestive heart failure	0	1481	0.0%	3962	2.3%	0.00
10	LF1 LF10	Angina pectoris	0	1481	0.0%	1773	1.0%	0.00
10	LF1 LF10	Hypothyroidism	0	1481	0.0%	3260	1.9%	0.00
10	LF1 LF10	Chronic kidney disease	0	1481	0.0%	1869	1.1%	0.00
10	LF1 LF10	Neuropathy	0	1481	0.0%	1964	1.1%	0.00
10	LF1 LF10	Osteoporosis	0	1481	0.0%	3233	1.8%	0.00
10	LF1 LF10	Primary open-angle glaucoma	0	1481	0.0%	2330	1.3%	0.00
10	LF1 LF10	Chronic bronchitis	0	1481	0.0%	2260	1.3%	0.00
10	LF1 LF10	Cervical spondylosis	0	1481	0.0%	1937	1.1%	0.00
10	LF1 LF10	Arthropathy	0	1481	0.0%	2168	1.2%	0.00

Definitions: Count, numbers of patients with that disease in that cluster; N cluster, total number of individuals within that cluster; Count prop, proportion of patients who have that disease within a cluster; Total disease, overall number of patients with that disease; Total dis. Prop, overall proportion that have that disease; O/E ratio, observed to expected ratio.

Supplementary Table S2. (continued) Description of clusters after Bayesian nonparametric model.

Cluster	Latent features	Read term	Count	N cluster	Count prop	Total disease	Total dis. Prop	O/E ratio
11	LF1 LF12	Cervical spondylosis	1369	1371	99.9%	1937	1.1%	90.41
11	LF1 LF12	Osteoarthritis	204	1371	14.9%	8954	5.1%	2.91
11	LF1 LF12	Irritable bowel syndrome	97	1371	7.1%	5019	2.9%	2.47
11	LF1 LF12	Chronic liver disease	72	1371	5.3%	4505	2.6%	2.04
11	LF1 LF12	Deafness	77	1371	5.6%	5261	3.0%	1.87
11	LF1 LF12	Senile cataract	108	1371	7.9%	7709	4.4%	1.79
11	LF1 LF12	Anxiety & other	51	1371	3.7%	4115	2.3%	1.59
11	LF1 LF12	Cancer	196	1371	14.3%	15,937	9.1%	1.57
11	LF1 LF12	Pure hypercholesterolaemia	69	1371	5.0%	5938	3.4%	1.49
11	LF1 LF12	High blood pressure	116	1371	8.5%	10,660	6.1%	1.39
11	LF1 LF12	Atrial fibrillation	76	1371	5.5%	7000	4.0%	1.39
11	LF1 LF12	Congestive heart failure	0	1371	0.0%	3962	2.3%	0.00
11	LF1 LF12	Chronic bronchitis	0	1371	0.0%	2260	1.3%	0.00
11	LF1 LF12	Angina pectoris	0	1371	0.0%	1773	1.0%	0.00
11	LF1 LF12	Primary open-angle glaucoma	0	1371	0.0%	2330	1.3%	0.00
11	LF1 LF12	Chronic kidney disease	0	1371	0.0%	1869	1.1%	0.00
11	LF1 LF12	Psoriasis or eczema	0	1371	0.0%	1975	1.1%	0.00
11	LF1 LF12	Obesity	0	1371	0.0%	2879	1.6%	0.00
11	LF1 LF12	Arthropathy	0	1371	0.0%	2168	1.2%	0.00
11	LF1 LF12	Intermittent claudication	0	1371	0.0%	2653	1.5%	0.00
11	LF1 LF12	Osteoporosis	0	1371	0.0%	3233	1.8%	0.00
11	LF1 LF12	Neuropathy	0	1371	0.0%	1964	1.1%	0.00
11	LF1 LF12	Hypothyroidism	0	1371	0.0%	3260	1.9%	0.00

Definitions: Count, numbers of patients with that disease in that cluster; N cluster, total number of individuals within that cluster; Count prop, proportion of patients who have that disease within a cluster; Total disease, overall number of patients with that disease; Total dis. Prop, overall proportion that have that disease; O/E ratio, observed to expected ratio.

Supplementary Table S2. (continued) Description of clusters after Bayesian nonparametric model.

Cluster	Latent features	Read term	Count	N cluster	Count prop	Total disease	Total dis. Prop	O/E ratio
12	LF1 LF11	Neuropathy	1350	1350	100.0%	1964	1.1%	89.30
12	LF1 LF11	Chronic liver disease	65	1350	4.8%	4505	2.6%	1.87
12	LF1 LF11	Irritable bowel syndrome	70	1350	5.2%	5019	2.9%	1.81
12	LF1 LF11	Osteoarthritis	116	1350	8.6%	8954	5.1%	1.68
12	LF1 LF11	Deafness	65	1350	4.8%	5261	3.0%	1.61
12	LF1 LF11	Senile cataract	95	1350	7.0%	7709	4.4%	1.60
12	LF1 LF11	High blood pressure	120	1350	8.9%	10,660	6.1%	1.46
12	LF1 LF11	Cancer	176	1350	13.0%	15,937	9.1%	1.43
12	LF1 LF11	Pure hypercholesterolaemia	65	1350	4.8%	5938	3.4%	1.42
12	LF1 LF11	Anxiety & other	42	1350	3.1%	4115	2.3%	1.33
12	LF1 LF11	Atrial fibrillation	66	1350	4.9%	7000	4.0%	1.22
12	LF1 LF11	Angina pectoris	0	1350	0.0%	1773	1.0%	0.00
12	LF1 LF11	Intermittent claudication	0	1350	0.0%	2653	1.5%	0.00
12	LF1 LF11	Chronic kidney disease	0	1350	0.0%	1869	1.1%	0.00
12	LF1 LF11	Primary open-angle glaucoma	0	1350	0.0%	2330	1.3%	0.00
12	LF1 LF11	Congestive heart failure	0	1350	0.0%	3962	2.3%	0.00
12	LF1 LF11	Chronic bronchitis	0	1350	0.0%	2260	1.3%	0.00
12	LF1 LF11	Obesity	0	1350	0.0%	2879	1.6%	0.00
12	LF1 LF11	Hypothyroidism	0	1350	0.0%	3260	1.9%	0.00
12	LF1 LF11	Cervical spondylosis	0	1350	0.0%	1937	1.1%	0.00
12	LF1 LF11	Psoriasis or eczema	0	1350	0.0%	1975	1.1%	0.00
12	LF1 LF11	Arthropathy	0	1350	0.0%	2168	1.2%	0.00
12	LF1 LF11	Osteoporosis	0	1350	0.0%	3233	1.8%	0.00

Definitions: Count, numbers of patients with that disease in that cluster; N cluster, total number of individuals within that cluster; Count prop, proportion of patients who have that disease within a cluster; Total disease, overall number of patients with that disease; Total dis. Prop, overall proportion that have that disease; O/E ratio, observed to expected ratio.

Supplementary Table S2. (continued) Description of clusters after Bayesian nonparametric model.

Cluster	Latent features	Read term	Count	N cluster	Count prop	Total disease	Total dis. Prop	O/E ratio
13	LF1 LF14	Angina pectoris	1253	1255	99.8%	1773	1.0%	98.76
13	LF1 LF14	Deafness	80	1255	6.4%	5261	3.0%	2.13
13	LF1 LF14	Atrial fibrillation	105	1255	8.4%	7000	4.0%	2.10
13	LF1 LF14	Irritable bowel syndrome	72	1255	5.7%	5019	2.9%	2.00
13	LF1 LF14	Anxiety & other	56	1255	4.5%	4115	2.3%	1.90
13	LF1 LF14	Pure hypercholesterolaemia	76	1255	6.1%	5938	3.4%	1.79
13	LF1 LF14	Osteoarthritis	113	1255	9.0%	8954	5.1%	1.76
13	LF1 LF14	Senile cataract	86	1255	6.9%	7709	4.4%	1.56
13	LF1 LF14	Chronic liver disease	48	1255	3.8%	4505	2.6%	1.49
13	LF1 LF14	Cancer	151	1255	12.0%	15,937	9.1%	1.32
13	LF1 LF14	High blood pressure	99	1255	7.9%	10,660	6.1%	1.30
13	LF1 LF14	Obesity	0	1255	0.0%	2879	1.6%	0.00
13	LF1 LF14	Psoriasis or eczema	0	1255	0.0%	1975	1.1%	0.00
13	LF1 LF14	Primary open-angle glaucoma	0	1255	0.0%	2330	1.3%	0.00
13	LF1 LF14	Hypothyroidism	0	1255	0.0%	3260	1.9%	0.00
13	LF1 LF14	Osteoporosis	0	1255	0.0%	3233	1.8%	0.00
13	LF1 LF14	Chronic kidney disease	0	1255	0.0%	1869	1.1%	0.00
13	LF1 LF14	Neuropathy	0	1255	0.0%	1964	1.1%	0.00
13	LF1 LF14	Cervical spondylosis	0	1255	0.0%	1937	1.1%	0.00
13	LF1 LF14	Intermittent claudication	0	1255	0.0%	2653	1.5%	0.00
13	LF1 LF14	Arthropathy	0	1255	0.0%	2168	1.2%	0.00
13	LF1 LF14	Chronic bronchitis	0	1255	0.0%	2260	1.3%	0.00
13	LF1 LF14	Congestive heart failure	0	1255	0.0%	3962	2.3%	0.00

Definitions: Count, numbers of patients with that disease in that cluster; N cluster, total number of individuals within that cluster; Count prop, proportion of patients who have that disease within a cluster; Total disease, overall number of patients with that disease; Total dis. Prop, overall proportion that have that disease; O/E ratio, observed to expected ratio.

Supplementary Table S2. (continued) Description of clusters after Bayesian nonparametric model.

Cluster	Latent features	Read term	Count	N cluster	Count prop	Total disease	Total dis. Prop	O/E ratio
14	LF1 LF13	Chronic kidney disease	1227	1228	99.9%	1869	1.1%	93.76
14	LF1 LF13	Atrial fibrillation	109	1228	8.9%	7000	4.0%	2.22
14	LF1 LF13	Deafness	81	1228	6.6%	5261	3.0%	2.20
14	LF1 LF13	Senile cataract	118	1228	9.6%	7709	4.4%	2.19
14	LF1 LF13	Cancer	195	1228	15.9%	15,937	9.1%	1.75
14	LF1 LF13	High blood pressure	104	1228	8.5%	10,660	6.1%	1.39
14	LF1 LF13	Irritable bowel syndrome	46	1228	3.7%	5019	2.9%	1.31
14	LF1 LF13	Pure hypercholesterolaemia	54	1228	4.4%	5938	3.4%	1.30
14	LF1 LF13	Osteoarthritis	68	1228	5.5%	8954	5.1%	1.08
14	LF1 LF13	Anxiety & other	31	1228	2.5%	4115	2.3%	1.08
14	LF1 LF13	Chronic liver disease	33	1228	2.7%	4505	2.6%	1.05
14	LF1 LF13	Osteoporosis	0	1228	0.0%	3233	1.8%	0.00
14	LF1 LF13	Angina pectoris	0	1228	0.0%	1773	1.0%	0.00
14	LF1 LF13	Psoriasis or eczema	0	1228	0.0%	1975	1.1%	0.00
14	LF1 LF13	Hypothyroidism	0	1228	0.0%	3260	1.9%	0.00
14	LF1 LF13	Arthropathy	0	1228	0.0%	2168	1.2%	0.00
14	LF1 LF13	Intermittent claudication	0	1228	0.0%	2653	1.5%	0.00
14	LF1 LF13	Cervical spondylosis	0	1228	0.0%	1937	1.1%	0.00
14	LF1 LF13	Primary open-angle glaucoma	0	1228	0.0%	2330	1.3%	0.00
14	LF1 LF13	Chronic bronchitis	0	1228	0.0%	2260	1.3%	0.00
14	LF1 LF13	Congestive heart failure	0	1228	0.0%	3962	2.3%	0.00
14	LF1 LF13	Neuropathy	0	1228	0.0%	1964	1.1%	0.00
14	LF1 LF13	Obesity	0	1228	0.0%	2879	1.6%	0.00

Definitions: Count, numbers of patients with that disease in that cluster; N cluster, total number of individuals within that cluster; Count prop, proportion of patients who have that disease within a cluster; Total disease, overall number of patients with that disease; Total dis. Prop, overall proportion that have that disease; O/E ratio, observed to expected ratio.

Supplementary Table S2. (continued) Description of clusters after Bayesian nonparametric model.

Cluster	Latent features	Read term	Count	N cluster	Count prop.	Total disease	Total dis. Prop.	O/E ratio
15	LF1 LF13 LF2	Chronic kidney disease	135	135	100.0%	1869	1.1%	93.84
15	LF1 LF13 LF2	Congestive heart failure	135	135	100.0%	3962	2.3%	44.27
15	LF1 LF13 LF2	Atrial fibrillation	45	135	33.3%	7000	4.0%	8.35
15	LF1 LF13 LF2	Senile cataract	17	135	12.6%	7709	4.4%	2.86
15	LF1 LF13 LF2	Cancer	26	135	19.3%	15,937	9.1%	2.12
15	LF1 LF13 LF2	Pure hypercholesterolaemia	9	135	6.7%	5938	3.4%	1.97
15	LF1 LF13 LF2	Osteoarthritis	10	135	7.4%	8954	5.1%	1.45
15	LF1 LF13 LF2	Irritable bowel syndrome	<7	135	3.7%	5019	2.9%	1.29
15	LF1 LF13 LF2	Deafness	<7	135	3.7%	5261	3.0%	1.23
15	LF1 LF13 LF2	High blood pressure	10	135	7.4%	10,660	6.1%	1.22
15	LF1 LF13 LF2	Chronic liver disease	<7	135	2.2%	4505	2.6%	0.87
15	LF1 LF13 LF2	Anxiety & other	<7	135	1.5%	4115	2.3%	0.63
15	LF1 LF13 LF2	Osteoporosis	0	135	0.0%	3233	1.8%	0.00
15	LF1 LF13 LF2	Arthropathy	0	135	0.0%	2168	1.2%	0.00
15	LF1 LF13 LF2	Cervical spondylosis	0	135	0.0%	1937	1.1%	0.00
15	LF1 LF13 LF2	Chronic bronchitis	0	135	0.0%	2260	1.3%	0.00
15	LF1 LF13 LF2	Intermittent claudication	0	135	0.0%	2653	1.5%	0.00
15	LF1 LF13 LF2	Angina pectoris	0	135	0.0%	1773	1.0%	0.00
15	LF1 LF13 LF2	Psoriasis or eczema	0	135	0.0%	1975	1.1%	0.00
15	LF1 LF13 LF2	Neuropathy	0	135	0.0%	1964	1.1%	0.00
15	LF1 LF13 LF2	Primary open-angle glaucoma	0	135	0.0%	2330	1.3%	0.00
15	LF1 LF13 LF2	Obesity	0	135	0.0%	2879	1.6%	0.00
15	LF1 LF13 LF2	Hypothyroidism	0	135	0.0%	3260	1.9%	0.00

Definitions: Count, numbers of patients with that disease in that cluster; N cluster, total number of individuals within that cluster; Count prop, proportion of patients who have that disease within a cluster; Total disease, overall number of patients with that disease; Total dis. Prop, overall proportion that have that disease; O/E ratio, observed to expected ratio.

Supplementary Table S2. (continued) Description of clusters after Bayesian nonparametric model.

Cluster	Latent features	Read term	Count	N cluster	Count prop.	Total disease	Total dis. Prop.	O/E ratio
16	LF1 LF8 LF2	Chronic bronchitis	126	126	100.0%	2260	1.3%	77.60
16	LF1 LF8 LF2	Congestive heart failure	125	126	99.2%	3962	2.3%	43.91
16	LF1 LF8 LF2	Atrial fibrillation	49	126	38.9%	7000	4.0%	9.74
16	LF1 LF8 LF2	Senile cataract	21	126	16.7%	7709	4.4%	3.79
16	LF1 LF8 LF2	Osteoarthritis	16	126	12.7%	8954	5.1%	2.49
16	LF1 LF8 LF2	Cancer	23	126	18.3%	15,937	9.1%	2.01
16	LF1 LF8 LF2	Pure hypercholesterolaemia	8	126	6.3%	5938	3.4%	1.88
16	LF1 LF8 LF2	Anxiety & other	<7	126	4.0%	4115	2.3%	1.69
16	LF1 LF8 LF2	Irritable bowel syndrome	<7	126	4.0%	5019	2.9%	1.39
16	LF1 LF8 LF2	Deafness	<7	126	3.2%	5261	3.0%	1.06
16	LF1 LF8 LF2	Chronic liver disease	<7	126	2.4%	4505	2.6%	0.93
16	LF1 LF8 LF2	High blood pressure	<7	126	2.4%	10,660	6.1%	0.39
16	LF1 LF8 LF2	Cervical spondylosis	0	126	0.0%	1937	1.1%	0.00
16	LF1 LF8 LF2	Osteoporosis	0	126	0.0%	3233	1.8%	0.00
16	LF1 LF8 LF2	Arthropathy	0	126	0.0%	2168	1.2%	0.00
16	LF1 LF8 LF2	Primary open-angle glaucoma	0	126	0.0%	2330	1.3%	0.00
16	LF1 LF8 LF2	Psoriasis or eczema	0	126	0.0%	1975	1.1%	0.00
16	LF1 LF8 LF2	Angina pectoris	0	126	0.0%	1773	1.0%	0.00
16	LF1 LF8 LF2	Intermittent claudication	0	126	0.0%	2653	1.5%	0.00
16	LF1 LF8 LF2	Hypothyroidism	0	126	0.0%	3260	1.9%	0.00
16	LF1 LF8 LF2	Chronic kidney disease	0	126	0.0%	1869	1.1%	0.00
16	LF1 LF8 LF2	Obesity	0	126	0.0%	2879	1.6%	0.00
16	LF1 LF8 LF2	Neuropathy	0	126	0.0%	1964	1.1%	0.00

Definitions: Count, numbers of patients with that disease in that cluster; N cluster, total number of individuals within that cluster; Count prop, proportion of patients who have that disease within a cluster; Total disease, overall number of patients with that disease; Total dis. Prop, overall proportion that have that disease; O/E ratio, observed to expected ratio.

Supplementary Table S2. (continued) Description of clusters after Bayesian nonparametric model.

Cluster	Latent features	Read term	Count	N cluster	Count prop.	Total disease	Total dis. Prop.	O/E ratio
17	LF1 V4 LF2	Osteoporosis	117	117	100.0%	3233	1.8%	54.25
17	LF1 V4 LF2	Congestive heart failure	117	117	100.0%	3962	2.3%	44.27
17	LF1 V4 LF2	Atrial fibrillation	29	117	24.8%	7000	4.0%	6.21
17	LF1 V4 LF2	Cancer	30	117	25.6%	15,937	9.1%	2.82
17	LF1 V4 LF2	Irritable bowel syndrome	9	117	7.7%	5019	2.9%	2.69
17	LF1 V4 LF2	Senile cataract	13	117	11.1%	7709	4.4%	2.53
17	LF1 V4 LF2	Anxiety & other	<7	117	4.3%	4115	2.3%	1.82
17	LF1 V4 LF2	Deafness	<7	117	4.3%	5261	3.0%	1.42
17	LF1 V4 LF2	Osteoarthritis	8	117	6.8%	8954	5.1%	1.34
17	LF1 V4 LF2	Pure hypercholesterolaemia	<7	117	2.6%	5938	3.4%	0.76
17	LF1 V4 LF2	High blood pressure	<7	117	4.3%	10,660	6.1%	0.70
17	LF1 V4 LF2	Chronic liver disease	<7	117	1.7%	4505	2.6%	0.67
17	LF1 V4 LF2	Chronic bronchitis	0	117	0.0%	2260	1.3%	0.00
17	LF1 V4 LF2	Angina pectoris	0	117	0.0%	1773	1.0%	0.00
17	LF1 V4 LF2	Intermittent claudication	0	117	0.0%	2653	1.5%	0.00
17	LF1 V4 LF2	Primary open-angle glaucoma	0	117	0.0%	2330	1.3%	0.00
17	LF1 V4 LF2	Obesity	0	117	0.0%	2879	1.6%	0.00
17	LF1 V4 LF2	Neuropathy	0	117	0.0%	1964	1.1%	0.00
17	LF1 V4 LF2	Chronic kidney disease	0	117	0.0%	1869	1.1%	0.00
17	LF1 V4 LF2	Hypothyroidism	0	117	0.0%	3260	1.9%	0.00
17	LF1 V4 LF2	Cervical spondylosis	0	117	0.0%	1937	1.1%	0.00
17	LF1 V4 LF2	Arthropathy	0	117	0.0%	2168	1.2%	0.00
17	LF1 V4 LF2	Psoriasis or eczema	0	117	0.0%	1975	1.1%	0.00

Definitions: Count, numbers of patients with that disease in that cluster; N cluster, total number of individuals within that cluster; Count prop, proportion of patients who have that disease within a cluster; Total disease, overall number of patients with that disease; Total dis. Prop, overall proportion that have that disease; O/E ratio, observed to expected ratio.

Supplementary Table S2. (continued) Description of clusters after Bayesian nonparametric model.

Cluster	Latent features	Read term	Count	N cluster	Count prop.	Total disease	Total dis. Prop.	O/E ratio
18	LF1 V3 LF2	Hypothyroidism	103	103	100.0%	3260	1.9%	53.80
18	LF1 V3 LF2	Congestive heart failure	103	103	100.0%	3962	2.3%	44.27
18	LF1 V3 LF2	Atrial fibrillation	35	103	34.0%	7000	4.0%	8.51
18	LF1 V3 LF2	Deafness	11	103	10.7%	5261	3.0%	3.56
18	LF1 V3 LF2	Chronic liver disease	8	103	7.8%	4505	2.6%	3.02
18	LF1 V3 LF2	Senile cataract	11	103	10.7%	7709	4.4%	2.43
18	LF1 V3 LF2	Cancer	19	103	18.4%	15,937	9.1%	2.03
18	LF1 V3 LF2	Irritable bowel syndrome	<7	103	4.9%	5019	2.9%	1.70
18	LF1 V3 LF2	Anxiety & other	<7	103	2.9%	4115	2.3%	1.24
18	LF1 V3 LF2	Osteoarthritis	<7	103	5.8%	8954	5.1%	1.14
18	LF1 V3 LF2	High blood pressure	<7	103	5.8%	10,660	6.1%	0.96
18	LF1 V3 LF2	Pure hypercholesterolaemia	<7	103	2.9%	5938	3.4%	0.86
18	LF1 V3 LF2	Chronic kidney disease	0	103	0.0%	1869	1.1%	0.00
18	LF1 V3 LF2	Psoriasis or eczema	0	103	0.0%	1975	1.1%	0.00
18	LF1 V3 LF2	Neuropathy	0	103	0.0%	1964	1.1%	0.00
18	LF1 V3 LF2	Chronic bronchitis	0	103	0.0%	2260	1.3%	0.00
18	LF1 V3 LF2	Intermittent claudication	0	103	0.0%	2653	1.5%	0.00
18	LF1 V3 LF2	Obesity	0	103	0.0%	2879	1.6%	0.00
18	LF1 V3 LF2	Osteoporosis	0	103	0.0%	3233	1.8%	0.00
18	LF1 V3 LF2	Primary open-angle glaucoma	0	103	0.0%	2330	1.3%	0.00
18	LF1 V3 LF2	Angina pectoris	0	103	0.0%	1773	1.0%	0.00
18	LF1 V3 LF2	Arthropathy	0	103	0.0%	2168	1.2%	0.00
18	LF1 V3 LF2	Cervical spondylosis	0	103	0.0%	1937	1.1%	0.00

Definitions: Count, numbers of patients with that disease in that cluster; N cluster, total number of individuals within that cluster; Count prop, proportion of patients who have that disease within a cluster; Total disease, overall number of patients with that disease; Total dis. Prop, overall proportion that have that disease; O/E ratio, observed to expected ratio.

Supplementary Table S2. (continued) Description of clusters after Bayesian nonparametric model.

Cluster	Latent features	Read term	Count	N cluster	Count prop.	Total disease	Total dis. Prop.	O/E ratio
19	LF1 LF6 LF2	Intermittent claudication	101	101	100.0%	2653	1.5%	66.11
19	LF1 LF6 LF2	Congestive heart failure	101	101	100.0%	3962	2.3%	44.27
19	LF1 LF6 LF2	Atrial fibrillation	32	101	31.7%	7000	4.0%	7.94
19	LF1 LF6 LF2	Deafness	10	101	9.9%	5261	3.0%	3.30
19	LF1 LF6 LF2	Senile cataract	14	101	13.9%	7709	4.4%	3.15
19	LF1 LF6 LF2	Irritable bowel syndrome	9	101	8.9%	5019	2.9%	3.11
19	LF1 LF6 LF2	Pure hypercholesterolaemia	9	101	8.9%	5938	3.4%	2.63
19	LF1 LF6 LF2	Osteoarthritis	10	101	9.9%	8954	5.1%	1.94
19	LF1 LF6 LF2	Cancer	17	101	16.8%	15,937	9.1%	1.85
19	LF1 LF6 LF2	Anxiety & other	<7	101	4.0%	4115	2.3%	1.69
19	LF1 LF6 LF2	High blood pressure	9	101	8.9%	10,660	6.1%	1.47
19	LF1 LF6 LF2	Chronic liver disease	<7	101	1.0%	4505	2.6%	0.39
19	LF1 LF6 LF2	Obesity	0	101	0.0%	2879	1.6%	0.00
19	LF1 LF6 LF2	Hypothyroidism	0	101	0.0%	3260	1.9%	0.00
19	LF1 LF6 LF2	Chronic bronchitis	0	101	0.0%	2260	1.3%	0.00
19	LF1 LF6 LF2	Neuropathy	0	101	0.0%	1964	1.1%	0.00
19	LF1 LF6 LF2	Psoriasis or eczema	0	101	0.0%	1975	1.1%	0.00
19	LF1 LF6 LF2	Arthropathy	0	101	0.0%	2168	1.2%	0.00
19	LF1 LF6 LF2	Cervical spondylosis	0	101	0.0%	1937	1.1%	0.00
19	LF1 LF6 LF2	Angina pectoris	0	101	0.0%	1773	1.0%	0.00
19	LF1 LF6 LF2	Chronic kidney disease	0	101	0.0%	1869	1.1%	0.00
19	LF1 LF6 LF2	Primary open-angle glaucoma	0	101	0.0%	2330	1.3%	0.00
19	LF1 LF6 LF2	Osteoporosis	0	101	0.0%	3233	1.8%	0.00

Definitions: Count, numbers of patients with that disease in that cluster; N cluster, total number of individuals within that cluster; Count prop, proportion of patients who have that disease within a cluster; Total disease, overall number of patients with that disease; Total dis. Prop, overall proportion that have that disease; O/E ratio, observed to expected ratio.

Supplementary Table S2. (continued) Description of clusters after Bayesian nonparametric model.

Cluster	Latent features	Read term	Count	N cluster	Count prop.	Total disease	Total dis. Prop.	O/E ratio
20	LF1 LF14 LF2	Angina pectoris	96	96	100.0%	1773	1.0%	98.92
20	LF1 LF14 LF2	Congestive heart failure	96	96	100.0%	3962	2.3%	44.27
20	LF1 LF14 LF2	Atrial fibrillation	25	96	26.0%	7000	4.0%	6.52
20	LF1 LF14 LF2	Senile cataract	14	96	14.6%	7709	4.4%	3.32
20	LF1 LF14 LF2	Deafness	8	96	8.3%	5261	3.0%	2.78
20	LF1 LF14 LF2	Irritable bowel syndrome	<7	96	6.3%	5019	2.9%	2.18
20	LF1 LF14 LF2	Cancer	17	96	17.7%	15,937	9.1%	1.95
20	LF1 LF14 LF2	Pure hypercholesterolaemia	<7	96	6.3%	5938	3.4%	1.85
20	LF1 LF14 LF2	Anxiety & other	<7	96	4.2%	4115	2.3%	1.78
20	LF1 LF14 LF2	Osteoarthritis	7	96	7.3%	8954	5.1%	1.43
20	LF1 LF14 LF2	Chronic liver disease	<7	96	3.1%	4505	2.6%	1.22
20	LF1 LF14 LF2	High blood pressure	<7	96	5.2%	10,660	6.1%	0.86
20	LF1 LF14 LF2	Obesity	0	96	0.0%	2879	1.6%	0.00
20	LF1 LF14 LF2	Arthropathy	0	96	0.0%	2168	1.2%	0.00
20	LF1 LF14 LF2	Hypothyroidism	0	96	0.0%	3260	1.9%	0.00
20	LF1 LF14 LF2	Osteoporosis	0	96	0.0%	3233	1.8%	0.00
20	LF1 LF14 LF2	Chronic kidney disease	0	96	0.0%	1869	1.1%	0.00
20	LF1 LF14 LF2	Neuropathy	0	96	0.0%	1964	1.1%	0.00
20	LF1 LF14 LF2	Chronic bronchitis	0	96	0.0%	2260	1.3%	0.00
20	LF1 LF14 LF2	Intermittent claudication	0	96	0.0%	2653	1.5%	0.00
20	LF1 LF14 LF2	Cervical spondylosis	0	96	0.0%	1937	1.1%	0.00
20	LF1 LF14 LF2	Primary open-angle glaucoma	0	96	0.0%	2330	1.3%	0.00
20	LF1 LF14 LF2	Psoriasis or eczema	0	96	0.0%	1975	1.1%	0.00

Definitions: Count, numbers of patients with that disease in that cluster; N cluster, total number of individuals within that cluster; Count prop, proportion of patients who have that disease within a cluster; Total disease, overall number of patients with that disease; Total dis. Prop, overall proportion that have that disease; O/E ratio, observed to expected ratio.

Supplementary Table S3. Comparison of disease prevalence between the three main cardiovascular clusters.

		Cluster 2 (LF1 LF2)		Cluster 15 (LF1 LF2 LF13)		Cluster 16 (LF1 LF2 LF8)		Proportions Comparison	
**Read term**	**Total dis. Prop**	**Count prop**	**O/E ratio**	**Count prop.**	**O/E ratio**	**Count prop.**	**O/E ratio**	**Cluster 15 vs Cluster 2**	**Cluster 16 vs Cluster 2**
Congestive heart failure	2.3%	98.0%	43.39	100.0%	44.27	99.2%	43.91	1.02	1.01
Atrial fibrillation	4.0%	28.1%	7.03	33.3%	8.35	38.9%	9.74	1.19	1.39
Senile cataract	4.4%	10.2%	2.31	12.6%	2.86	16.7%	3.79	1.24	1.64
Deafness	3.0%	6.1%	2.02	3.7%	1.23	3.2%	1.06	0.61	0.52
Irritable bowel syndrome	2.9%	5.1%	1.78	3.7%	1.29	4.0%	1.39	0.73	0.78
Cancer	9.1%	14.3%	1.57	19.3%	2.12	18.3%	2.01	1.35	1.28
Osteoarthritis	5.1%	6.6%	1.30	7.4%	1.45	12.7%	2.49	1.12	1.92
Anxiety & other	2.3%	2.7%	1.16	1.5%	0.63	4.0%	1.69	0.55	1.46
Chronic liver disease	2.6%	3.0%	1.15	2.2%	0.87	2.4%	0.93	0.75	0.80
High blood pressure	6.1%	5.5%	0.90	7.4%	1.22	2.4%	0.39	1.36	0.44
Pure hypercholesterolaemia	3.4%	2.8%	0.82	6.7%	1.97	6.3%	1.88	2.39	2.28
Osteoporosis	1.8%	0.0%	0.00	0.0%	0.00	0.0%	0.00	0.00	0.00
Obesity	1.6%	0.0%	0.00	0.0%	0.00	0.0%	0.00	0.00	0.00
Cervical spondylosis	1.1%	0.0%	0.00	0.0%	0.00	0.0%	0.00	0.00	0.00
Angina pectoris	1.0%	0.0%	0.00	0.0%	0.00	0.0%	0.00	0.00	0.00
Arthropathy	1.2%	0.0%	0.00	0.0%	0.00	0.0%	0.00	0.00	0.00
Intermittent claudication	1.5%	0.0%	0.00	0.0%	0.00	0.0%	0.00	0.00	0.00
Psoriasis or eczema	1.1%	0.0%	0.00	0.0%	0.00	0.0%	0.00	0.00	0.00
Neuropathy	1.1%	0.0%	0.00	0.0%	0.00	0.0%	0.00	0.00	0.00
Hypothyroidism	1.9%	0.0%	0.00	0.0%	0.00	0.0%	0.00	0.00	0.00
Chronic kidney disease	1.1%	0.0%	0.00	100.0%	93.84	0.0%	0.00	∞	0.00
Primary open-angle glaucoma	1.3%	0.0%	0.00	0.0%	0.00	0.0%	0.00	0.00	0.00
Chronic bronchitis	1.3%	0.0%	0.00	0.0%	0.00	100.0%	77.60	0.00	∞

Definitions: Total dis. Prop, overall proportion that have that disease; Count prop, proportion of patients who have that disease within a cluster; O/E ratio, observed to expected ratio.

Supplementary Table S4. Demographic characteristics of 175,383 T2DM patients at index date (first NIAD prescription) stratified by cluster, first 20 clusters.

	Cluster 1 (N=147,816)	Cluster 2 (N=2766)	Cluster 3 (N=2572)	Cluster 4 (N=2350)	Cluster 5 (N=2252)
Index Age (mean (SD))	60.0 (14.3)	69.5 (11.6)	60.2 (13.9)	67.9 (12.2)	53.0 (12.9)
Smoking (%)					
Current	49,633 (33.7)	1256 (45.6)	847 (33.2)	788 (33.7)	733 (32.7)
Never	72,228 (49.1)	1108 (40.2)	1302 (51.0)	1210 (51.8)	1084 (48.4)
Previous	25,260 (17.2)	392 (14.2)	403 (15.8)	340 (14.5)	423 (18.9)
Unknown	<7	<7	<7	<7	<7
Alcohol consumption (%)					
Current	6157 (4.5)	126 (4.9)	122 (5.2)	84 (3.8)	79 (3.8)
Never	32,387 (23.6)	623 (24.0)	623 (26.5)	663 (30.1)	487 (23.6)
Previous	98,626 (71.9)	1846 (71.1)	1608 (68.3)	1454 (66.0)	1499 (72.6)
Unknown	24 (0.0)	<7	<7	<7	<7
BMI (mean (SD))	32.5 (7.0)	32.5 (6.7)	32.8 (7.4)	30.7 (6.4)	38.43 (7.1)
Comorbidities ever-before (%)					
Cancer	37,362 (25.3)	796 (28.8)	636 (24.7)	752 (32.0)	478 (21.2)
Hypothyroidism	11,658 (7.9)	255 (9.2)	<7	287 (12.2)	183 (8.1)
Pure hypercholesterolaemia	23,126 (15.6)	565 (20.4)	394 (15.3)	474 (20.2)	295 (13.1)
Obesity	17,007 (11.5)	328 (11.9)	333 (12.9)	220 (9.4)	<7
Anxiety & other	25,080 (17.0)	422 (15.3)	459 (17.8)	440 (18.7)	424 (18.8)
Neuropathy	1050 (0.7)	38 (1.4)	10 (0.4)	26 (1.1)	12 (0.5)
Primary open-angle glaucoma	5052 (3.4)	115 (4.2)	101 (3.9)	114 (4.9)	55 (2.4)
Senile cataract	6651 (4.5)	240 (8.7)	111 (4.3)	179 (7.6)	40 (1.8)
Deafness	11,740 (7.9)	320 (11.6)	205 (8.0)	235 (10.0)	120 (5.3)
High blood pressure	54,867 (37.1)	1468 (53.1)	916 (35.6)	1089 (46.3)	793 (35.2)
Angina pectoris	9024 (6.1)	464 (16.8)	165 (6.4)	227 (9.7)	92 (4.1)
Atrial fibrillation	9910 (6.7)	531 (19.2)	191 (7.4)	200 (8.5)	94 (4.2)
Congestive heart failure	4170 (2.8)	<7	87 (3.4)	82 (3.5)	43 (1.9)
Intermittent claudication	4447 (3.0)	198 (7.2)	61 (2.4)	95 (4.0)	44 (2.0)
Chronic bronchitis	2300 (1.6)	109 (3.9)	24 (0.9)	69 (2.9)	27 (1.2)
Irritable bowel syndrome	14,196 (9.6)	225 (8.1)	248 (9.6)	280 (11.9)	211 (9.4)
Chronic liver disease	3724 (2.5)	37 (1.3)	51 (2.0)	74 (3.1)	41 (1.8)
Chronic kidney disease	893 (0.6)	34 (1.2)	16 (0.6)	24 (1.0)	7 (0.3)
Psoriasis or eczema	8611 (5.8)	179 (6.5)	107 (4.2)	151 (6.4)	148 (6.6)
Osteoarthritis	27,809 (18.8)	802 (29.0)	458 (17.8)	757 (32.2)	364 (16.2)
Arthropathy	8131 (5.5)	224 (8.1)	139 (5.4)	211 (9.0)	103 (4.6)
Cervical spondylosis	10,498 (7.1)	294 (10.6)	181 (7.0)	280 (11.9)	126 (5.6)
Osteoporosis	6005 (4.1)	154 (5.6)	103 (4.0)	<7	56 (2.5)

Supplementary Table S4. (continued) Demographic characteristics of 175,383 T2DM patients at index date (first NIAD prescription) stratified by cluster, first 20 clusters.

	Cluster 6 (N=1871)	Cluster 7 (N=1795)	Cluster 8 (N=1532)	Cluster 9 (N=1496)	Cluster 10 (N=1481)
Index Age (mean (SD))	64.7 (11.7)	64.8 (11.9)	61.8 (11.9)	66.1 (10.1)	56.5 (12.9)
Smoking (%)					
Current	705 (37.8)	672 (37.7)	551 (36.1)	777 (51.9)	562 (38.1)
Never	526 (28.2)	885 (49.7)	753 (49.3)	165 (11.0)	572 (38.7)
Previous	633 (34.0)	225 (12.6)	224 (14.7)	554 (37.0)	342 (23.2)
Unknown	<7	<7	<7	<7	<7
Alcohol consumption (%)					
Current	84 (4.9)	59 (3.5)	65 (4.5)	112 (7.8)	68 (4.9)
Never	363 (21.0)	366 (22.0)	361 (24.9)	321 (22.5)	290 (21.0)
Previous	1280 (74.1)	1239 (74.5)	1023 (70.6)	993 (69.6)	1026 (74.1)
Unknown	<7	<7	<7	<7	<7
BMI (mean (SD))	30.7 (6.2)	31.12 (6.3)	33.4 (6.9)	33.0 (7.1)	33.8 (7.2)
Comorbidities ever-before (%)					
Cancer	496 (26.5)	480 (26.7)	382 (24.9)	404 (27.0)	354 (23.9)
Hypothyroidism	138 (7.4)	160 (8.9)	159 (10.4)	140 (9.4)	141 (9.5)
Pure hypercholesterolaemia	409 (21.9)	322 (17.9)	287 (18.7)	284 (19.0)	225 (15.2)
Obesity	158 (8.4)	159 (8.9)	238 (15.5)	228 (15.2)	184 (12.4)
Anxiety & other	330 (17.6)	252 (14.0)	296 (19.3)	354 (23.7)	277 (18.7)
Neuropathy	22 (1.2)	15 (0.8)	8 (0.5)	12 (0.8)	8 (0.5)
Primary open-angle glaucoma	84 (4.5)	<7	55 (3.6)	57 (3.8)	39 (2.6)
Senile cataract	109 (5.8)	89 (5.0)	79 (5.2)	105 (7.0)	52 (3.5)
Deafness	183 (9.8)	155 (8.6)	160 (10.4)	166 (11.1)	114 (7.7)
High blood pressure	786 (42.0)	739 (41.2)	683 (44.6)	661 (44.2)	509 (34.4)
Angina pectoris	229 (12.2)	120 (6.7)	112 (7.3)	196 (13.1)	79 (5.3)
Atrial fibrillation	171 (9.1)	107 (6.0)	112 (7.3)	152 (10.2)	73 (4.9)
Congestive heart failure	90 (4.8)	38 (2.1)	41 (2.7)	95 (6.4)	29 (2.0)
Intermittent claudication	<7	55 (3.1)	42 (2.7)	100 (6.7)	32 (2.2)
Chronic bronchitis	42 (2.2)	25 (1.4)	25 (1.6)	<7	24 (1.6)
Irritable bowel syndrome	167 (8.9)	174 (9.7)	205 (13.4)	200 (13.4)	160 (10.8)
Chronic liver disease	35 (1.9)	35 (1.9)	36 (2.3)	42 (2.8)	34 (2.3)
Chronic kidney disease	14 (0.7)	11 (0.6)	<7	7 (0.5)	<7
Psoriasis or eczema	140 (7.5)	130 (7.2)	113 (7.4)	117 (7.8)	<7
Osteoarthritis	438 (23.4)	363 (20.2)	470 (30.7)	421 (28.1)	249 (16.8)
Arthropathy	115 (6.1)	116 (6.5)	<7	146 (9.8)	87 (5.9)
Cervical spondylosis	190 (10.2)	161 (9.0)	179 (11.7)	184 (12.3)	96 (6.5)
Osteoporosis	88 (4.7)	86 (4.8)	65 (4.2)	113 (7.6)	55 (3.7)

Supplementary Table S4. (continued) Demographic characteristics of 175,383 T2DM patients at index date (first NIAD prescription) stratified by cluster, first 20 clusters.

	Cluster 11 (N=1371)	Cluster 12 (N=1350)	Cluster 13 (N=1255)	Cluster 14 (N=1228)	Cluster 15 (N=135)
Index Age (mean (SD))	62.2 (11.7)	61.6 (12.6)	62.2 (11.0)	69.1 (10.9)	71.8 (8.8)
Smoking (%)					
Current	454 (33.2)	519 (38.7)	534 (42.6)	479 (39.1)	57 (42.5)
Never	701 (51.2)	588 (43.8)	511 (40.7)	550 (44.9)	54 (40.3)
Previous	213 (15.6)	234 (17.4)	209 (16.7)	196 (16.0)	23 (17.2)
Unknown	<7	<7	<7	<7	<7
Alcohol consumption (%)					
Current	62 (4.8)	57 (4.5)	53 (4.5)	51 (4.5)	<7
Never	339 (26.1)	301 (24.0)	275 (23.2)	304 (26.6)	27 (21.4)
Previous	900 (69.2)	895 (71.4)	857 (72.3)	788 (68.9)	93 (73.8)
Unknown	<7	<7	<7	<7	<7
BMI (mean (SD))	32.5 (6.4)	32.2 (6.5)	32.7 (6.4)	31.7 (6.6)	31.0 (6.4)
Comorbidities ever-before (%)					
Cancer	393 (28.7)	388 (28.7)	314 (25.0)	317 (25.8)	29 (21.5)
Hypothyroidism	115 (8.4)	105 (7.8)	121 (9.6)	122 (9.9)	9 (6.7)
Pure hypercholesterolaemia	273 (19.9)	268 (19.9)	263 (21.0)	226 (18.4)	27 (20.0)
Obesity	206 (15.0)	171 (12.7)	173 (13.8)	152 (12.4)	16 (11.9)
Anxiety & other	275 (20.1)	320 (23.7)	230 (18.3)	184 (15.0)	13 (9.6)
Neuropathy	11 (0.8)	<7	15 (1.2)	12 (1.0)	<7
Primary open-angle glaucoma	57 (4.2)	71 (5.3)	43 (3.4)	64 (5.2)	8 (5.9)
Senile cataract	57 (4.2)	76 (5.6)	56 (4.5)	90 (7.3)	15 (11.1)
Deafness	117 (8.5)	154 (11.4)	135 (10.8)	106 (8.6)	14 (10.4)
High blood pressure	584 (42.6)	521 (38.6)	555 (44.2)	659 (53.7)	70 (51.9)
Angina pectoris	107 (7.8)	113 (8.4)	<7	118 (9.6)	36 (26.7)
Atrial fibrillation	76 (5.5)	106 (7.9)	89 (7.1)	131 (10.7)	27 (20.0)
Congestive heart failure	26 (1.9)	38 (2.8)	40 (3.2)	100 (8.1)	<7
Intermittent claudication	41 (3.0)	47 (3.5)	67 (5.3)	65 (5.3)	15 (11.1)
Chronic bronchitis	19 (1.4)	38 (2.8)	28 (2.2)	25 (2.0)	<7
Irritable bowel syndrome	178 (13.0)	173 (12.8)	141 (11.2)	111 (9.0)	15 (11.1)
Chronic liver disease	32 (2.3)	45 (3.3)	46 (3.7)	22 (1.8)	<7
Chronic kidney disease	<7	8 (0.6)	7 (0.6)	<7	<7
Psoriasis or eczema	90 (6.6)	87 (6.4)	74 (5.9)	81 (6.6)	7 (5.2)
Osteoarthritis	388 (28.3)	340 (25.2)	281 (22.4)	336 (27.4)	35 (25.9)
Arthropathy	124 (9.0)	104 (7.7)	89 (7.1)	89 (7.2)	10 (7.4)
Cervical spondylosis	<7	152 (11.3)	132 (10.5)	127 (10.3)	16 (11.9)
Osteoporosis	78 (5.7)	64 (4.7)	73 (5.8)	57 (4.6)	<7 (2.2)

Supplementary Table S4. (continued) Demographic characteristics of 175,383 T2DM patients at index date (first NIAD prescription) stratified by cluster, first 20 clusters.

	Cluster 16 (N=126)	Cluster 17 (N=117)	Cluster 18 (N=103)	Cluster 19 (N=101)	Cluster 20 (N=96)
Index Age (mean (SD))	68.7 (8.5)	74.7 (9.4)	71.1 (11.5)	70.1 (10.9)	66.4 (10.4)
Smoking (%)					
Current	64 (50.8)	51 (43.6)	41 (40.2)	38 (37.6)	48 (50.0)
Never	18 (14.3)	44 (37.6)	47 (46.1)	33 (32.7)	37 (38.5)
Previous	44 (34.9)	22 (18.8)	14 (13.7)	30 (29.7)	11 (11.5)
Unknown	<7	<7	<7	<7	<7
Alcohol consumption (%)					
Current	13 (10.8)	<7	<7	<7	<7
Never	14 (11.7)	36 (33.3)	23 (24.7)	15 (16.7)	18 (20.2)
Previous	93 (77.5)	69 (63.9)	69 (74.2)	72 (80.0)	69 (77.5)
Unknown	<7	<7	<7	<7	<7
BMI (mean (SD))	33.7 (7.5)	30.1 (6.3)	32.1 (6.1)	31.4 (5.9)	32.0 (5.8)
Comorbidities ever-before (%)					
Cancer	37 (29.4)	31 (26.5)	28 (27.2)	22 (21.8)	25 (26.0)
Hypothyroidism	11 (8.7)	10 (8.5)	<7	11 (10.9)	<7 (6.2)
Pure hypercholesterolaemia	24 (19.0)	20 (17.1)	16 (15.5)	25 (24.8)	17 (17.7)
Obesity	21 (16.7)	9 (7.7)	8 (7.8)	11 (10.9)	10 (10.4)
Anxiety & other	27 (21.4)	19 (16.2)	21 (20.4)	12 (11.9)	15 (15.6)
Neuropathy	<7	<7	<7	<7	<7
Primary open-angle glaucoma	7 (5.6)	7 (6.0)	<7	11 (10.9)	<7
Senile cataract	9 (7.1)	18 (15.4)	<7	11 (10.9)	<7
Deafness	16 (12.7)	20 (17.1)	12 (11.7)	12 (11.9)	9 (9.4)
High blood pressure	67 (53.2)	74 (63.2)	50 (48.5)	46 (45.5)	44 (45.8)
Angina pectoris	23 (18.3)	21 (17.9)	14 (13.6)	21 (20.8)	<7
Atrial fibrillation	13 (10.3)	24 (20.5)	17 (16.5)	15 (14.9)	<7 (6.2)
Congestive heart failure	<7	<7	<7	<7	<7
Intermittent claudication	10 (7.9)	<7	<7	<7	<7 (6.2)
Chronic bronchitis	<7	<7	<7	<7	<7
Irritable bowel syndrome	15 (11.9)	17 (14.5)	11 (10.7)	<7	10 (10.4)
Chronic liver disease	<7	<7	<7	<7	<7
Chronic kidney disease	<7	<7	<7	<7	<7
Psoriasis or eczema	9 (7.1)	8 (6.8)	7 (6.8)	9 (8.9)	<7
Osteoarthritis	44 (34.9)	46 (39.3)	28 (27.2)	25 (24.8)	31 (32.3)
Arthropathy	20 (15.9)	8 (6.8)	9 (8.7)	9 (8.9)	11 (11.5)
Cervical spondylosis	13 (10.3)	16 (13.7)	9 (8.7)	20 (19.8)	14 (14.6)
Osteoporosis	9 (7.1)	<7	<7	<7	<7

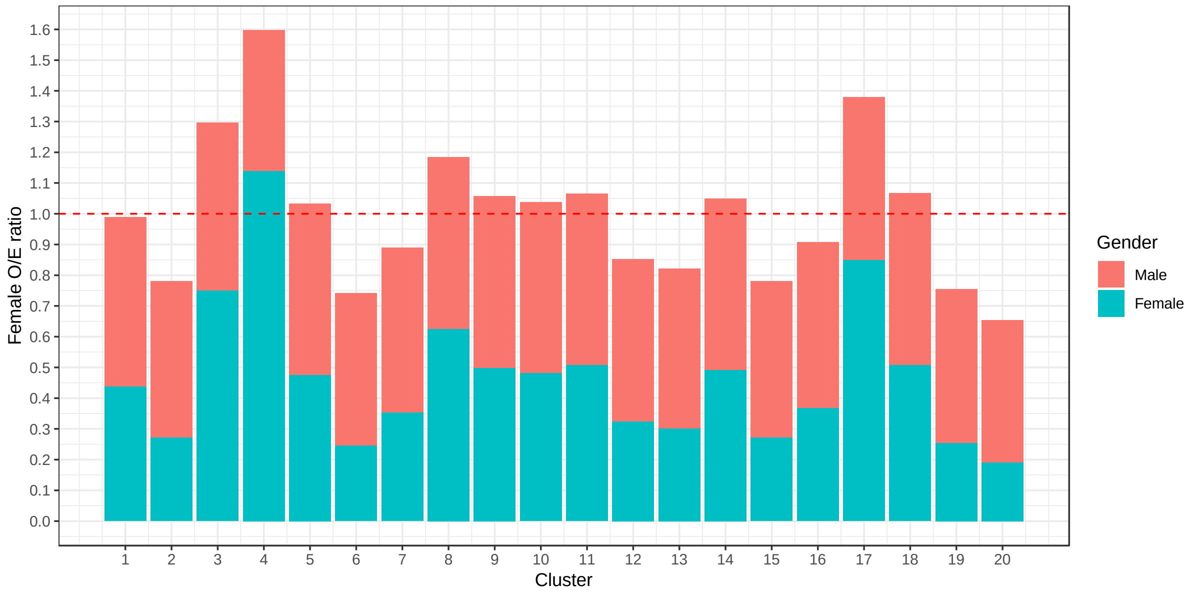

Supplementary Figure S1. O/E ratios for women for the first 20 clusters. The bars above O/E ratios 1.0 indicate that that specific cluster had a higher proportion of females than males. The colours represent the proportion of male and females in each cluster.

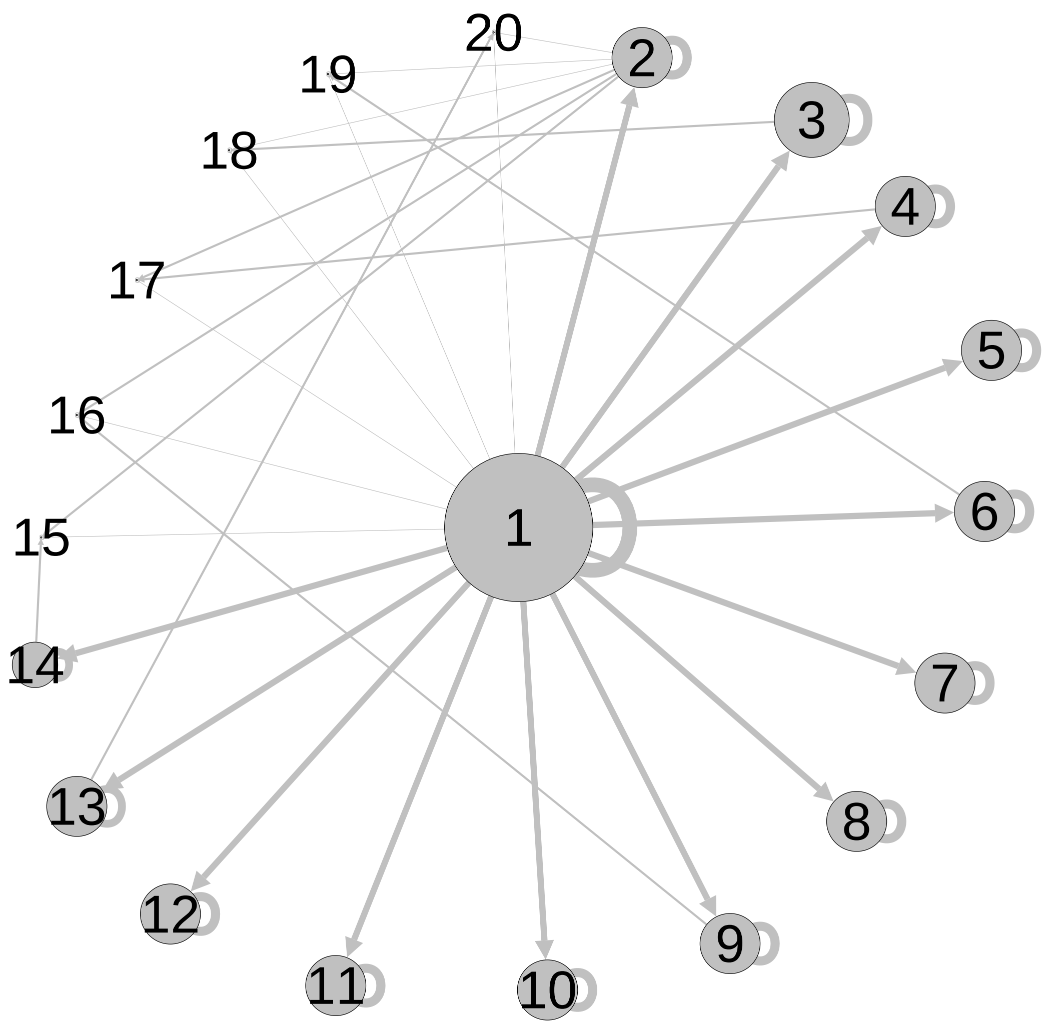

Supplementary Figure S2. Network representation of the transitions between first 20 clusters. Nodes represent clusters, edges indicate the transition between two given clusters, and the node size corresponds to the logarithm of the size (number of patients in that node at any time). The nodes of clusters 15 to 20 had a low proportion of patients, thus a very small node size.
